# Tree-Based Machine Learning for Diagnostic Classification of Dengue Fever Using Routine Hematological Parameters: A Secondary Analysis of a Publicly Available Dataset

**DOI:** 10.3390/diagnostics16182966

**Published:** 2026-09-14

**Authors:** Zeynep Burcin Yilmaz, Zeynep Kucukakcali, Sami Akbulut

**Affiliations:** 1 Department of Infectious Diseases and Clinical Microbiology, Faculty of Medicine, Inonu University, Malatya 44280, Türkiye; burcin.yilmaz@inonu.edu.tr; 2 Department of Biostatistics and Medical Informatics, Faculty of Medicine, Inonu University, Malatya 44280, Türkiye; zeynep.tunc@inonu.edu.tr; 3 Department of Surgery and Liver Transplantation, Faculty of Medicine, Inonu University, Malatya 44280, Türkiye; 4 Department of Public Health, Faculty of Medicine, Inonu University, Malatya 44280, Türkiye

**Keywords:** dengue fever, hematological parameters, machine learning, tree-based models, logistic regression, diagnostic classification

## Abstract

**Background**: Dengue fever remains a major global health problem, and early diagnosis is challenging where confirmatory testing is limited. Machine-learning studies using routine hematological data have focused mainly on discrimination, whereas calibration, decision-analytic performance, interpretability, and robust validation have received less attention. This study aimed to develop and compare tree-based machine-learning models for dengue classification, benchmark them against L2-penalized logistic regression (LR), and evaluate discrimination, calibration, potential decision-analytic benefit, and interpretability. **Methods**: This retrospective secondary analysis used an open-access dataset from Bangladesh comprising 1523 patients, 18 demographic and hematological predictors, and a binary dengue test outcome. Data were divided into stratified training (80%) and test (20%) sets. The Synthetic Minority Over-sampling Technique was applied only within the training workflow. Random Forest (RF), XGBoost, and LightGBM were optimized using Optuna with stratified five-fold cross-validation. L2-penalized LR was evaluated using the same predictors and training–test partition. Held-out test-set performance was assessed using AUROC, AUPRC, accuracy, sensitivity, specificity, predictive values, F1-score, and Brier score. Calibration, decision curve analysis, SHAP values, and permutation importance were also examined. **Results**: LightGBM, RF, and XGBoost yielded AUROCs of 0.709, 0.704, and 0.702, respectively, indicating closely similar discrimination. The primary SMOTE-trained LR yielded a numerically lower AUROC of 0.608 (95% CI: 0.536–0.677) and a higher Brier score of 0.310 than the tree-based models (0.174–0.176); however, in sensitivity analysis without SMOTE, the LR AUROC increased numerically to 0.655 and the Brier score decreased to 0.194. At the training-derived threshold of 0.558, LightGBM achieved a sensitivity of 0.914 and a specificity of 0.458, reflecting a high-sensitivity, low-specificity profile. The LightGBM calibration curve suggested closer agreement in the low-to-moderate predicted-probability range, with greater deviation at higher probabilities. Decision curve analysis suggested potential net benefit across a range of threshold probabilities but did not establish clinical utility. Platelet count, monocyte percentage, and neutrophil percentage were consistently among the leading predictors across the tree-based models. **Conclusions**: Tree-based models showed moderate discrimination, with high sensitivity but limited specificity, and yielded numerically higher AUROCs and lower Brier scores than the primary SMOTE-trained LR within this internal-validation framework. They should not replace etiological testing or be used as standalone diagnostic tools; their observed operating characteristics are more compatible with a potential adjunctive screening or triage-support role. External and prospective validation across independent populations and settings is required before clinical use or superiority over simpler statistical models can be established.

## 1. Introduction

Dengue fever is one of the most important mosquito-borne viral diseases worldwide and remains a major threat to public health in tropical and subtropical regions [1,2]. It is caused by the dengue virus (DENV), a flavivirus with four antigenically distinct serotypes, and is transmitted primarily by *Aedes aegypti* and *Aedes albopictus* mosquitoes [2]. Epidemiological estimates suggest that nearly two-fifths of the global population are at risk of dengue, with approximately 390 million infections, 500,000 hospitalizations, and 20,000 deaths occurring annually worldwide [1,2,3]. Recent studies emphasize that dengue incidence has risen markedly over the past two decades, driven by rapid urbanization, climate-sensitive ecological change, expanding vector habitats, human mobility, and the cocirculation of multiple serotypes [1,2,4]. As a result, dengue is increasingly recognized as an expanding global health problem rather than a disease confined to traditionally endemic settings [1,2].

Clinically, dengue has a broad spectrum of presentations [2]. Many infections are asymptomatic or self-limited, yet a substantial proportion present as acute febrile illness with high fever, headache, retro-orbital pain, myalgia, arthralgia, rash, nausea, and vomiting [2]. In some patients, illness progresses to a critical phase characterized by plasma leakage, bleeding manifestations, and organ dysfunction [2]. Severe dengue may involve shock, metabolic derangement, hepatic injury, renal impairment, encephalitis, or myocarditis [2]. Available evidence suggests that this clinical heterogeneity reflects complex interactions between viral factors and host responses, including antibody-dependent enhancement, NS1-related endothelial injury, cytokine dysregulation, and immune-mediated vascular dysfunction [1,2]. These clinical and biological complexities make early recognition particularly important, because timely supportive care allows appropriate clinical monitoring and management and may reduce the risk of adverse outcomes [2,5].

Despite advances in laboratory medicine, dengue diagnosis remains challenging in real-world practice [2,5,6]. Clinical manifestations overlap extensively with those of other acute febrile illnesses, including malaria, chikungunya, and nonspecific viral syndromes, especially in endemic and resource-limited settings [5]. Recent literature reviews have shown that this diagnostic uncertainty may lead to inappropriate treatment, including unnecessary antimalarial or antibiotic use, delayed recognition of severe disease, and suboptimal patient management [5]. Laboratory confirmation is therefore essential, but currently available tests each have important limitations [2,5,6]. Molecular assays such as RT-qPCR remain highly informative during the early viremic phase, yet their use depends on laboratory infrastructure, trained personnel, and appropriate timing of sample collection [2,5,7]. NS1 antigen assays offer practical advantages in the first days of illness, particularly in low-resource settings, but their sensitivity may be reduced in secondary infection and later presentation [2,5,7]. Serologic tests such as IgM and IgG ELISA are helpful in later phases of disease, although cross-reactivity with other flaviviruses and phase-dependent interpretation remain important concerns [2,5,7,8]. Together, these limitations help explain why dengue diagnosis often remains imperfect, delayed, or context-dependent [5].

Against this background, interest in artificial intelligence (AI), machine learning (ML), and deep learning (DL) approaches for dengue has expanded substantially [9,10]. A systematic review of dengue modeling has shown that computational methods have been developed across three broad domains: diagnostic modeling, epidemic forecasting, and intervention-oriented modeling [10]. More recently, a diagnosis-focused systematic review identified 32 eligible studies and 48 distinct ML/DL algorithms, highlighting the methodological diversity of this field [9]. Support vector machines and random forest (RF) models have been frequently used, while boosted tree methods and neural-network approaches have also demonstrated promising performance [9,10]. Separate studies have applied ML to outbreak prediction using climate variables [11,12,13,14], to prevalence forecasting using weather-integrated models [4,15,16], and to severity prediction using clinical and laboratory parameters [17,18,19]. Together, these studies suggest that data-driven models have potential applications in diagnostic support, risk stratification, and public health decision-making [9,10,17,18,19].

Nevertheless, important gaps remain. Many published ML studies differ substantially in case definitions, predictor sets, outcome targets, and validation strategies [9,10]. Some focus on ecological forecasting rather than patient-level diagnosis, while others use symptom-based or laboratory-based data but lack broad external validation or consistent generalizability across populations [10,20]. Moreover, systematic reviews have emphasized the heterogeneity of algorithms and study designs, making direct comparison difficult and limiting immediate clinical translation [9,10]. These limitations support the evaluation of simple and clinically accessible predictors that are routinely available at the point of care [20,21,22].

The rationale for using routine hematological parameters extends beyond their accessibility and low cost. Dengue infection is associated with characteristic hematological alterations resulting from viral–host interactions, immune activation, bone-marrow suppression, peripheral cellular destruction, and changes in platelet and leukocyte dynamics. Thrombocytopenia and leukopenia are commonly observed during dengue infection, while changes in hematocrit may reflect plasma leakage and disease progression. These interrelated hematological alterations provide a biological and clinical rationale for evaluating multivariable patterns in routinely available blood parameters rather than considering individual markers in isolation. In this context, complete blood count (CBC) parameters are particularly attractive because they are widely available, inexpensive, and biologically relevant to dengue pathophysiology [1,2,21,22,23]. Nirob et al. [23] introduced a publicly available hematological dataset comprising 1523 patients from Bangladesh, including routinely collected demographic and hematological variables together with binary dengue test outcomes. Using this same source dataset, Tuhin et al. [22] subsequently developed an ensemble-based ML framework with extensive preprocessing and SHAP/LIME-based interpretation, whereas Jahan et al. [21] investigated an interpretable Tree-Augmented Naive Bayes approach. Importantly, previous studies using the same publicly available Bangladeshi dataset leave several methodological questions unresolved. Jahan et al. evaluated an interpretable Tree-Augmented Naive Bayes framework against several conventional classifiers using 10-fold cross-validation and reported discrimination and classification metrics, but did not extend model evaluation to probability calibration or decision-analytic net benefit. Their approach also required discretization of continuous hematological variables, and the authors acknowledged the absence of external validation. In contrast, Tuhin et al. developed the more complex DengueStackX-19 stacking ensemble and incorporated SHAP and LIME explanations, reporting substantially higher classification performance after evaluating multiple resampling strategies. However, their reported analytical workflow describes class balancing during preprocessing before the subsequent 80/20 data partition, and does not clearly indicate that resampling was restricted to the training folds, raising a potential risk of information leakage and optimistic performance estimation. Moreover, neither study provided an integrated assessment combining discrimination, probability calibration, decision-analytic net benefit, threshold selection independent of the held-out test set, and complementary global feature-importance analyses. These gaps provide a specific rationale for re-examining the same dataset under a more explicitly leakage-conscious and multidimensional evaluation framework. Although these studies demonstrated the feasibility of ML-based classification using routine hematological data, a comprehensive comparison of widely used tree-based algorithms that integrates discrimination, calibration, threshold-dependent performance, potential decision-analytic benefit, and complementary explainability analyses remains warranted.

Accordingly, the primary question of the present study was not whether one tree-based algorithm is intrinsically superior to another, but how reliably dengue can be classified using routine demographic and hematological information alone when model development and evaluation are conducted within a leakage-conscious and multidimensional analytical framework. RF, XGBoost, and LightGBM were selected as representative tree-based ensemble approaches because they are well suited to structured tabular data, can capture nonlinear relationships and interactions among hematological variables, and permit complementary assessment of feature importance. These models were used to examine the consistency of predictive performance across related algorithmic architectures rather than to identify a universally optimal classifier. Beyond discrimination, the study was designed to characterize probability calibration, threshold-dependent operating characteristics, potential decision-analytic benefit, and model interpretability using complementary SHAP and permutation-importance analyses. Particular emphasis was placed on preventing information leakage by isolating the held-out test set from resampling, hyperparameter optimization, and threshold selection. Thus, the intended contribution is not a directly deployable standalone diagnostic tool, but a rigorously evaluated benchmark for understanding the potential and limitations of routine hematological data for dengue screening and triage and for informing the development of future externally validated decision-support models.

## 2. Materials and Methods

### 2.1. Study Design and Data Source

This retrospective secondary ML study was conducted using a publicly available open-access hematological dataset originally described by Nirob et al. [23] and deposited in the Mendeley Data repository (DOI: 10.17632/6fsrsk3mb8.2). The source dataset comprises 1523 patient records collected at Jamalpur Medical College Hospital, Jamalpur, Bangladesh. The original dataset contains 19 attributes in total; in the present analysis, 18 demographic and hematological variables were used as candidate predictors, while the remaining variable was used as the binary classification outcome. The predictor variables included age, sex, hemoglobin, neutrophil percentage (%), lymphocyte percentage (%), monocyte percentage (%), eosinophil percentage (%), red blood cell (RBC) count, hematocrit (HCT, %), mean corpuscular volume (MCV, fL), mean corpuscular hemoglobin (MCH, pg), mean corpuscular hemoglobin concentration (MCHC, g/dL), red cell distribution width-coefficient of variation (RDW-CV, %), platelet count (/cumm), mean platelet volume (MPV, fL), platelet distribution width (PDW, %), plateletcrit (PCT, %), and total white blood cell (WBC) count (/cumm). The outcome was encoded as dengue-positive = 1 and dengue-negative = 0, consistent with the positive/negative test-result classification provided in the source dataset.

### 2.2. Data Preprocessing

The dataset contained no missing values; therefore, no missing-data imputation was required. The dataset was divided into training and test sets using a stratified split that preserved the original class distribution, with 80% of observations allocated to training (*n* = 1218) and 20% to testing (*n* = 305). The overall dataset comprised 1523 patients, including 1042 dengue-positive (68.4%) and 481 dengue-negative (31.6%) cases. Following stratified 80/20 splitting, the training set included 1218 patients (833 dengue-positive [68.4%] and 385 dengue-negative [31.6%]), whereas the held-out test set comprised 305 patients (209 dengue-positive [68.5%] and 96 dengue-negative [31.5%]). The test set was held out from the entire model development process and was not used during hyperparameter optimization, resampling, or threshold determination. This hold-out set was reserved exclusively for final performance evaluation.

### 2.3. Handling of Class Imbalance

To reduce the adverse effect of class imbalance on model performance, the Synthetic Minority Over-sampling Technique (SMOTE) was applied during model development. To prevent data leakage, SMOTE was applied only to the training data and, within each cross-validation iteration, only to the training subset of the corresponding fold. Validation and test subsets were never exposed to synthetic observations at any stage. For this purpose, SMOTE was embedded within an imbalanced-learn pipeline, allowing automatic and isolated resampling within each fold of cross-validation.

### 2.4. Model Development

Three tree-based ensemble learning algorithms were used for classification because of their strong empirical performance in structured clinical data and their ability to capture nonlinear relationships: RF, Extreme Gradient Boosting (XGBoost), and Light Gradient Boosting Machine (LightGBM).

RF is an ensemble method based on bootstrap aggregation (bagging), in which a large number of decision trees are trained independently on bootstrap samples of the training data, while each split considers a random subset of predictors. Final classification is determined by majority voting across trees. Compared with a single decision tree, this method is more resistant to overfitting and can model complex nonlinear interactions between variables.

XGBoost is an optimized implementation of the gradient boosting framework. In contrast to RF, trees are trained sequentially rather than independently, with each new tree constructed to reduce the residual errors of the preceding ensemble. The algorithm incorporates L1 (reg_alpha) and L2 (reg_lambda) regularization to reduce overfitting and includes optimized tree construction and pruning mechanisms to improve computational efficiency.

LightGBM is a gradient boosting algorithm developed by Microsoft. Similar to XGBoost, it relies on sequential boosting; however, it differs by using a leaf-wise tree growth strategy rather than a level-wise strategy and by applying histogram-based split finding, which improves training speed and reduces memory consumption. These properties contribute to computational efficiency when applying LightGBM to structured tabular data. Because the leaf-wise strategy may increase model complexity by generating unbalanced deep trees, parameters such as num_leaves and max_depth were carefully constrained. All three algorithms are tree-based and therefore do not require feature scaling such as normalization or standardization and can capture nonlinear relationships and complex interactions among predictors. They also provide mechanisms for assessing feature importance, supporting subsequent model-interpretability analyses. These characteristics supported the selection of tree-based approaches for both predictive performance and clinical interpretability.

In addition to the three tree-based machine-learning algorithms, a multivariable logistic regression (LR) model was developed as a conventional statistical comparator. The LR model was constructed using the same 18 demographic and hematological predictors and the identical stratified 80:20 training–test partition used for RF, XGBoost, and LightGBM, thereby enabling direct comparison under the same evaluation framework. Model development was restricted to the training set, and the held-out test set remained completely independent until final performance evaluation. Continuous predictors were standardized using parameters estimated exclusively from the training data. Consistent with the primary modeling framework, class imbalance was addressed using SMOTE exclusively within the training data, without introducing synthetic observations into the held-out test set. The final LR model was evaluated using the same performance metrics as the tree-based models, including AUROC, AUPRC, sensitivity, specificity, and Brier score, with calibration additionally assessed to compare the agreement between predicted probabilities and observed outcomes.

### 2.5. Hyperparameter Optimization

Hyperparameter optimization for each algorithm was performed using Optuna, a Bayesian optimization framework. Tree-structured Parzen Estimator (TPE) sampling was used as the search strategy, and a Median Pruner was used for early stopping of unpromising trials. For each model, stratified 5-fold cross-validation (StratifiedKFold, k = 5) was conducted on the training set, and 30 trials were run to maximize the area under the receiver operating characteristic curve (AUROC). To ensure reproducibility, a fixed random seed (random_state = 42) was used throughout all stages of the analysis.

For RF, the following hyperparameters were tuned: number of trees (n_estimators: 100–350), maximum tree depth (max_depth: 4–14), minimum number of samples required to split a node (min_samples_split: 2–12), minimum number of samples required at a terminal node (min_samples_leaf: 1–10), and the number of features considered at each split (max_features: sqrt or log2).

For XGBoost, the tuned hyperparameters included number of trees (n_estimators: 100–350), maximum depth (max_depth: 3–8), learning rate (learning_rate: 0.02–0.2, logarithmic scale), row subsampling ratio (subsample: 0.6–1.0), column subsampling ratio (colsample_bytree: 0.6–1.0), minimum child weight (min_child_weight: 1–8), and L1/L2 regularization coefficients (reg_alpha and reg_lambda: 0.001–5, logarithmic scale).

For LightGBM, the tuned hyperparameters included number of trees (n_estimators: 100–350), maximum depth (max_depth: 3–10), learning rate (learning_rate: 0.02–0.2, logarithmic scale), number of leaves (num_leaves: 15–80), row and column subsampling ratios (subsample and colsample_bytree: 0.6–1.0), minimum number of samples required in a leaf (min_child_samples: 5–40), and L1/L2 regularization coefficients (reg_alpha and reg_lambda: 0.001–5, logarithmic scale). The optimal hyperparameter configuration identified for each algorithm was subsequently used to refit the corresponding model on the full training set, with SMOTE applied within the predefined training pipeline, to obtain the final models.

The LR model was fitted using L2 regularization (penalty = ‘l2’) with the default inverse regularization strength (C = 1.0). The lbfgs solver was used with a maximum of 5000 iterations (max_iter = 5000), an intercept term was included (fit_intercept = True), the convergence tolerance was 1 × 10^−4^, and no class weights were applied (class_weight = None). A fixed random seed of 42 was specified. Predictor scaling and SMOTE (random_state = 42) were incorporated within the imbalanced-learn pipeline to prevent information leakage.

### 2.6. Model Performance Evaluation

The final models were evaluated on the held-out test set using the following performance metrics: AUROC, area under the precision-recall curve (AUPRC), accuracy, sensitivity, specificity, positive predictive value (PPV), negative predictive value (NPV), F1-score, and Brier score. Comparative model performance was also examined visually using receiver operating characteristic (ROC) curves and precision-recall curves. To quantify sampling uncertainty in the held-out test-set performance estimates, 95% confidence intervals were calculated using 2000 nonparametric bootstrap resamples of the test set. Bootstrap confidence intervals were obtained for AUROC, AUPRC, sensitivity, specificity, and Brier score. For threshold-dependent measures, confidence intervals were calculated separately using the default classification threshold of 0.50 and the model-specific thresholds derived exclusively from out-of-fold predictions in the training set; these thresholds were kept fixed during bootstrap resampling.

### 2.7. Decision-Threshold Optimization

Because the default classification threshold of 0.50 may not be optimal in imbalanced datasets, a model-specific decision threshold was determined for each model. Threshold optimization was performed exclusively using out-of-fold predicted probabilities generated from the training set during stratified cross-validation, and the threshold maximizing Youden’s J statistic (sensitivity + specificity − 1) was selected. The test set was not involved in threshold selection and was used only for final evaluation after threshold optimization.

### 2.8. Calibration and Decision Curve Analysis

To assess the agreement between predicted probabilities and observed event frequencies, calibration curves were generated and Brier scores were calculated. To evaluate the potential decision-analytic benefit of the models, decision curve analysis (DCA) was performed by estimating the net benefit across a range of threshold probabilities and comparing it with the treat-all and treat-none strategies. No post-hoc probability correction or recalibration was performed after model fitting with SMOTE. Accordingly, calibration curves, Brier scores, and decision curve analyses were based on the uncorrected predicted probabilities generated by the SMOTE-trained models when applied to the original, non-resampled held-out test set.

### 2.9. Model Explainability

To improve the interpretability of the model with the numerically highest AUROC, two complementary explainability approaches were applied: (i) SHAP (SHapley Additive exPlanations) values were used to quantify the contribution of each variable to individual model predictions using a game-theoretic framework; and (ii) permutation importance analysis was used to assess global feature importance based on predictive performance by measuring the reduction in AUROC after randomly shuffling the values of each variable, using 20 repetitions.

### 2.10. Statistical Software

All analyses were performed using Python (version 3.12.13). Data manipulation was conducted with pandas and NumPy; ML model development and evaluation with scikit-learn; gradient boosting algorithms with XGBoost and LightGBM; class imbalance correction with imbalanced-learn; hyperparameter optimization with Optuna; explainability analyses with SHAP; and visualization with Matplotlib and Seaborn. A fixed random seed (42) was used throughout the analytical workflow to enhance reproducibility.

### 2.11. Study Protocol, Ethical Considerations, and Funding

This study was conducted as a secondary analysis of a previously published, publicly available dengue fever dataset using machine-learning methods. No new patient recruitment, intervention, or additional data collection was performed. The original dataset was collected from patients admitted to Jamalpur Medical College Hospital, Jamalpur, Bangladesh, and was anonymized to protect participant privacy. According to the original publication, informed consent was obtained from each participant or their legal guardian, and the original study was approved by the board of Jamalpur Medical College Hospital on 10 February 2025. The present secondary analysis was conducted in accordance with the ethical principles of the Declaration of Helsinki, including its 2024 revision [24], and with applicable institutional and national regulations governing research involving human participants. Ethical approval for the present study was obtained from the Inonu University Institutional Review Board for Non-Interventional Clinical Research (Date: 14 July 2026; Approval No: 10748). Because the present study involved no new participant recruitment or contact and used only anonymized, publicly available data, no additional informed consent was obtained for the secondary analysis. The study was reported in accordance with the TRIPOD + AI statement for prediction model studies using regression or machine-learning methods [25], with relevant STROBE principles additionally considered for reporting aspects related to the underlying observational data source [26]. This research received no external funding.

## 3. Results

### 3.1. Comparison of Model Performance Using the Default Threshold (0.50)

**Figure 1:**
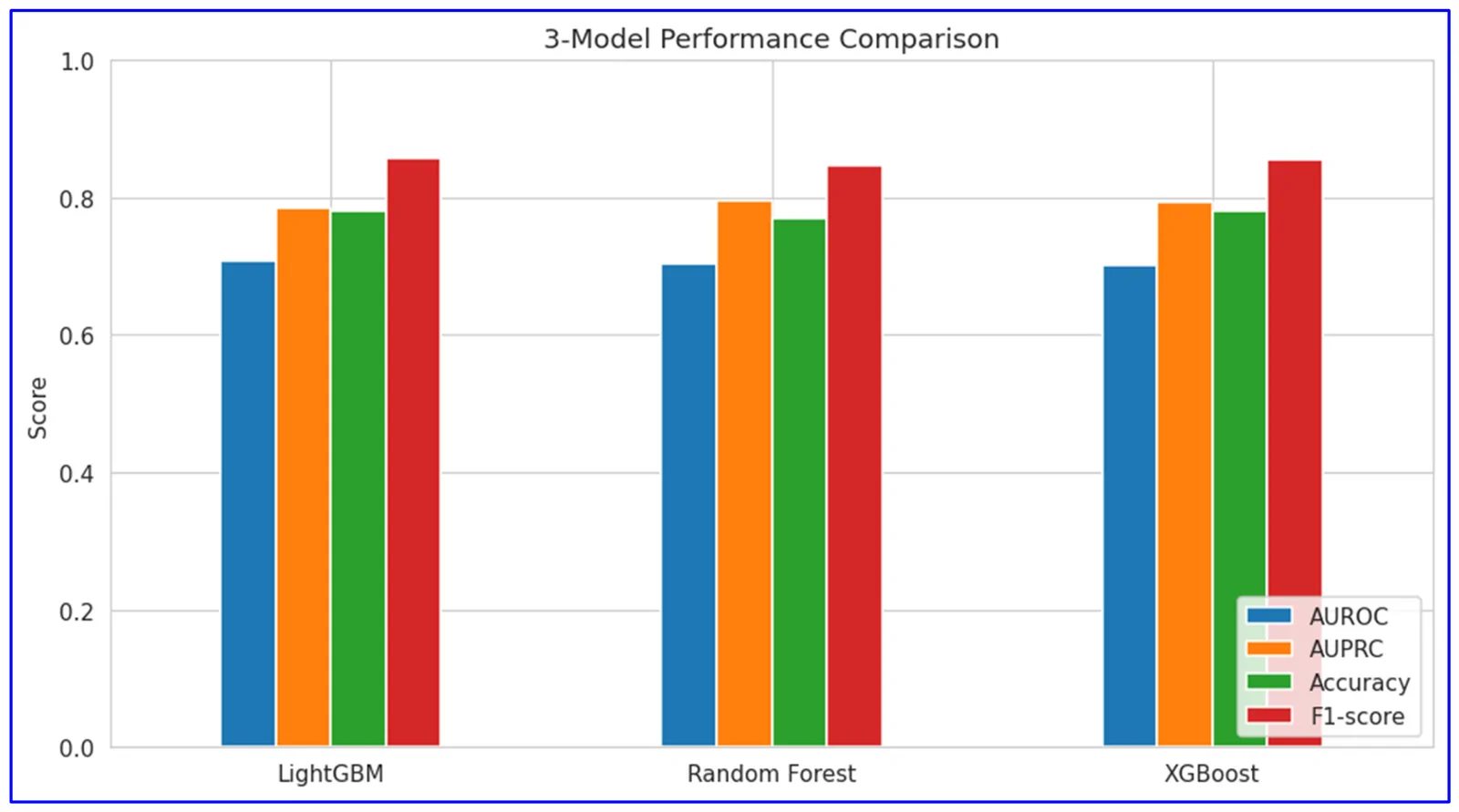
Comparative performance of the three tree-based classifiers on the held-out test set at the default classification threshold of 0.50. Bars represent AUROC, AUPRC, accuracy, and F1-score for LightGBM, RF, and XGBoost. AUROC and AUPRC are threshold-independent measures, whereas accuracy and F1 score were calculated using the default classification threshold of 0.50.

Table 1 shows that performance metrics were obtained on the held-out test set using the default classification threshold of 0.50. A comparative summary of AUROC, AUPRC, accuracy, and F1-score across the three models is shown in Figure 1. LightGBM yielded the numerically highest AUROC (0.709), followed by RF (0.704) and XGBoost (0.702). However, the AUROC point estimates were closely similar across the three models, with absolute differences below 0.01. These findings indicate broadly comparable discrimination and should not be interpreted as evidence of superiority of any individual algorithm.

Table 1 also shows that LightGBM yielded the highest sensitivity (0.962) and the highest F1-score (0.857), whereas RF achieved the highest AUPRC (0.796). Accuracy was similar across models, ranging from 0.771 to 0.780. However, the higher sensitivity of LightGBM at the default threshold was accompanied by lower specificity (0.385) compared with RF (0.438) and XGBoost (0.417), indicating a higher false-positive classification rate for LightGBM at the default threshold.

### 3.2. Determination of Youden J–Derived Thresholds

Table 2 shows the model-specific thresholds derived by maximizing Youden’s J statistic using only cross-validated predictions from the training set. The Youden J–derived threshold was 0.545 for RF (Youden’s J = 0.348), 0.537 for XGBoost (Youden’s J = 0.355), and 0.558 for LightGBM (Youden’s J = 0.352). The performance metrics obtained after applying these thresholds to the test set are presented in Table 2. Application of the Youden J–derived thresholds increased specificity across all three models, accompanied by a reduction in sensitivity. For LightGBM, specificity increased from 0.385 to 0.458, whereas sensitivity decreased from 0.962 to 0.914. For RF, specificity increased from 0.438 to 0.469, with sensitivity decreasing from 0.923 to 0.895. For XGBoost, specificity increased from 0.417 to 0.438, while sensitivity decreased from 0.947 to 0.923. Because AUROC, AUPRC, and Brier score are threshold-independent metrics, these measures remained unchanged after application of the Youden J–derived thresholds. Overall, application of these thresholds increased specificity at the cost of reduced sensitivity across all three models.

### 3.3. Detailed Evaluation of the LightGBM Model

**Figure 2:**
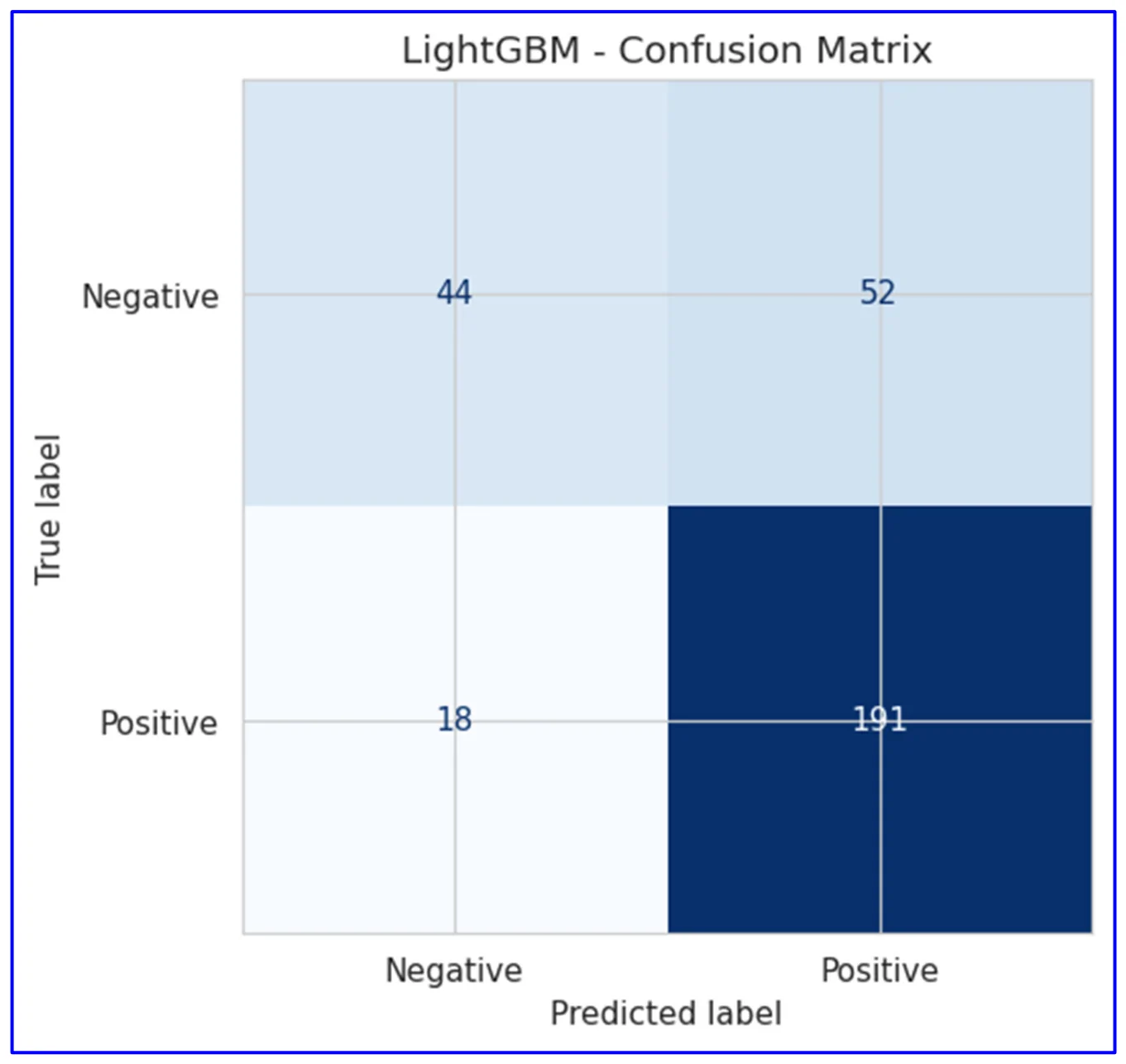
Confusion matrix of the LightGBM model on the held-out test set using the training-derived optimized threshold of 0.558. Rows represent the observed dengue test status and columns represent the predicted class. The model classified 44 patients as true negatives, 52 as false positives, 18 as false negatives, and 191 as true positives.

**Figure 3:**
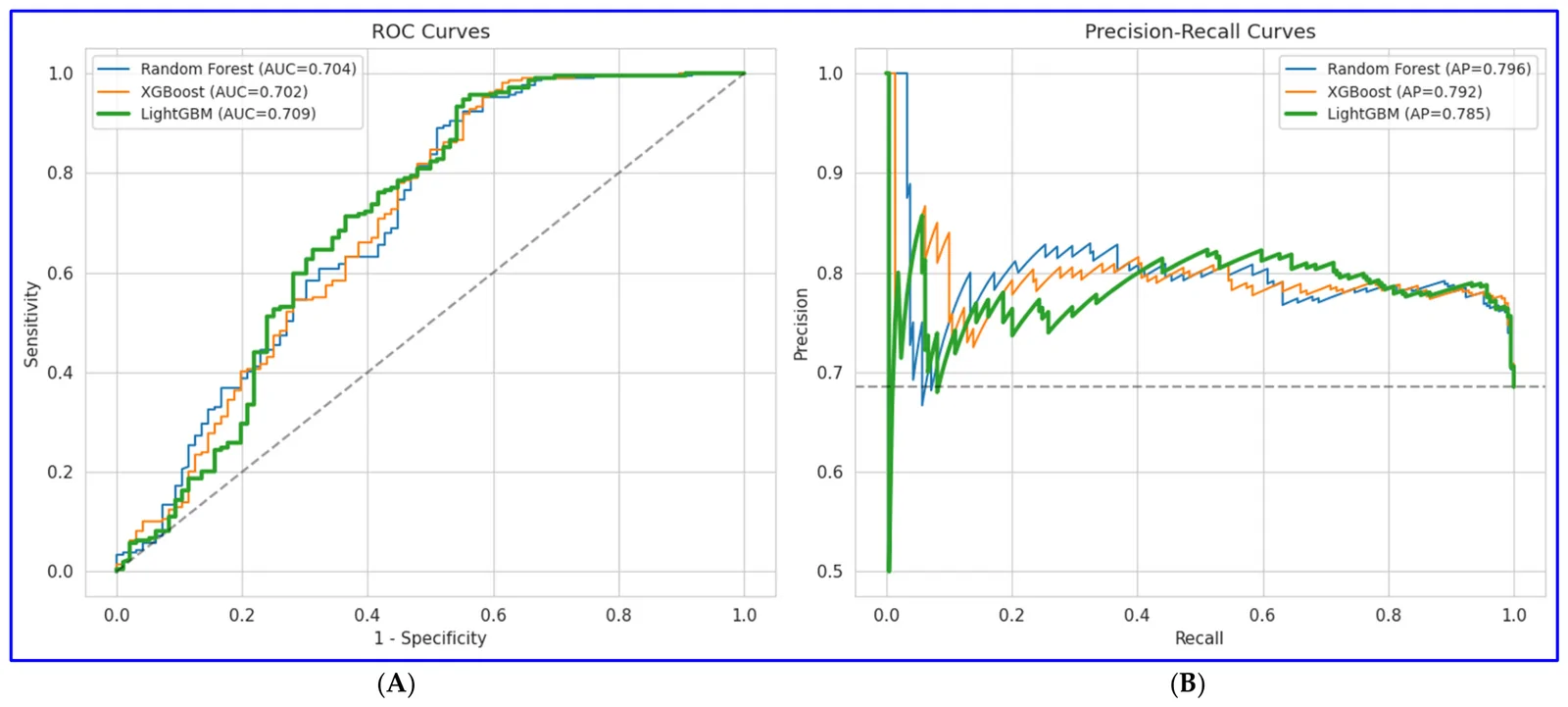
Discrimination performance of the tree-based classifiers on the held-out test set. (**A**) Receiver operating characteristic curves for RF, XGBoost, and LightGBM, with the corresponding areas under the curve (left). The diagonal dashed line represents chance-level discrimination. (**B**) Precision–recall curves with the corresponding areas under the precision–recall curve (right). The horizontal dashed line represents the prevalence of dengue positivity in the test set.

Figure 2 shows that, using the optimized threshold of 0.558, the LightGBM model correctly classified 235 (77.0%) of the 305 patients in the test set. Of the 96 patients with a negative dengue result, 44 were correctly classified as true negatives and 52 were misclassified as false positives. Of the 209 patients with a positive dengue result, 191 were correctly classified as true positives and 18 were misclassified as false negatives. Because LightGBM yielded the numerically highest AUROC among the three models, it was selected as the representative model for detailed downstream evaluation; however, this selection was based on its numerical ranking and should not be interpreted as evidence of statistical superiority over RF or XGBoost. Figure 3 demonstrates that the ROC and precision-recall curves of all three models followed closely similar trajectories, and no model demonstrated a clearly dominant pattern across the full range of thresholds.

### 3.4. Calibration Performance and Decision Curve Analysis

**Figure 4:**
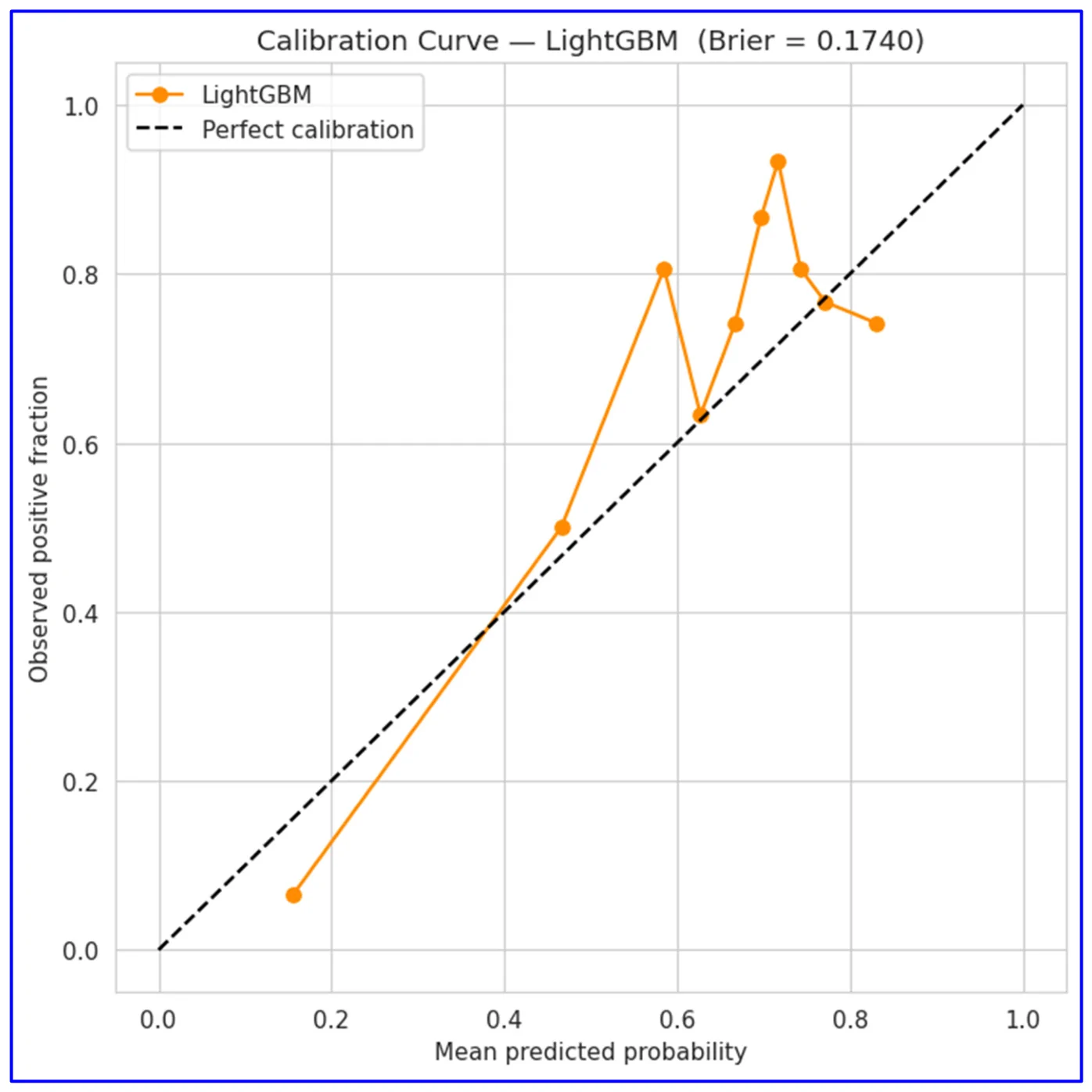
Calibration of LightGBM-predicted dengue probabilities on the held-out test set. Observed dengue-positive proportions are plotted against the mean predicted probabilities across calibration bins. The dashed diagonal line represents perfect calibration. The Brier score of the LightGBM model was 0.174.

**Figure 5:**
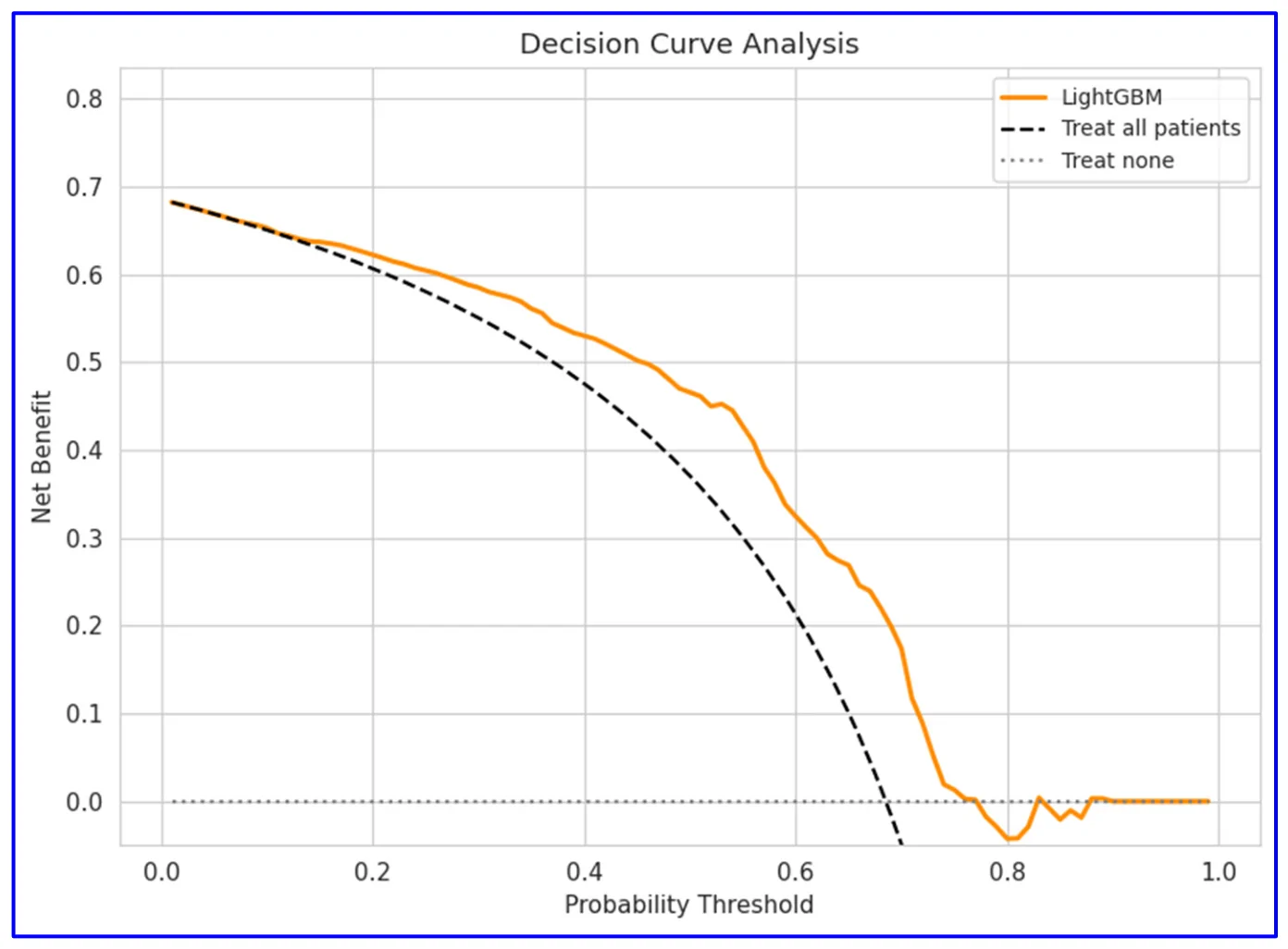
Decision curve analysis of the LightGBM model on the held-out test set. Net benefit is plotted across a range of threshold probabilities. The solid line represents the LightGBM model, the dashed line represents the strategy of classifying all patients as dengue positive, and the dotted line represents the strategy of classifying no patients as dengue positive.

Figure 4 suggests that the LightGBM model showed closer agreement with the ideal calibration line within the low-to-moderate predicted-probability range (approximately 0.15–0.65), with greater deviation at higher predicted probabilities (>0.65). The Brier score for LightGBM was 0.174. Figure 5 demonstrates that, in the Decision Curve Analysis, across an approximate threshold-probability range of 0.10–0.68, the LightGBM model provided greater net benefit than the treat-all strategy; however, this range represents the observed decision-analytic behavior of the model rather than a prespecified clinically optimal threshold interval.

### 3.5. Comparison with L2-Penalized LR

To provide a conventional statistical comparator, an L2-penalized LR model was developed using the same 18 demographic and hematological predictors and evaluated under the same training–test framework as the tree-based models. Performance was assessed on the held-out test set using both the default probability threshold of 0.50 and a training-derived optimized threshold of 0.108. Table 3 shows that the LR model achieved an AUROC of 0.608 (95% CI: 0.536–0.677) and an AUPRC of 0.753 (95% CI: 0.688–0.820). At the default threshold, sensitivity and specificity were 0.742 and 0.438, respectively. Application of the training-derived optimized threshold increased sensitivity to 0.837 but reduced specificity to 0.302. The Brier score was 0.310 (95% CI: 0.266–0.356). Overall, the primary SMOTE-trained LR showed lower discrimination than RF, XGBoost, and LightGBM, which achieved AUROC values of 0.704, 0.702, and 0.709, respectively (Table 1).

**Figure 6:**
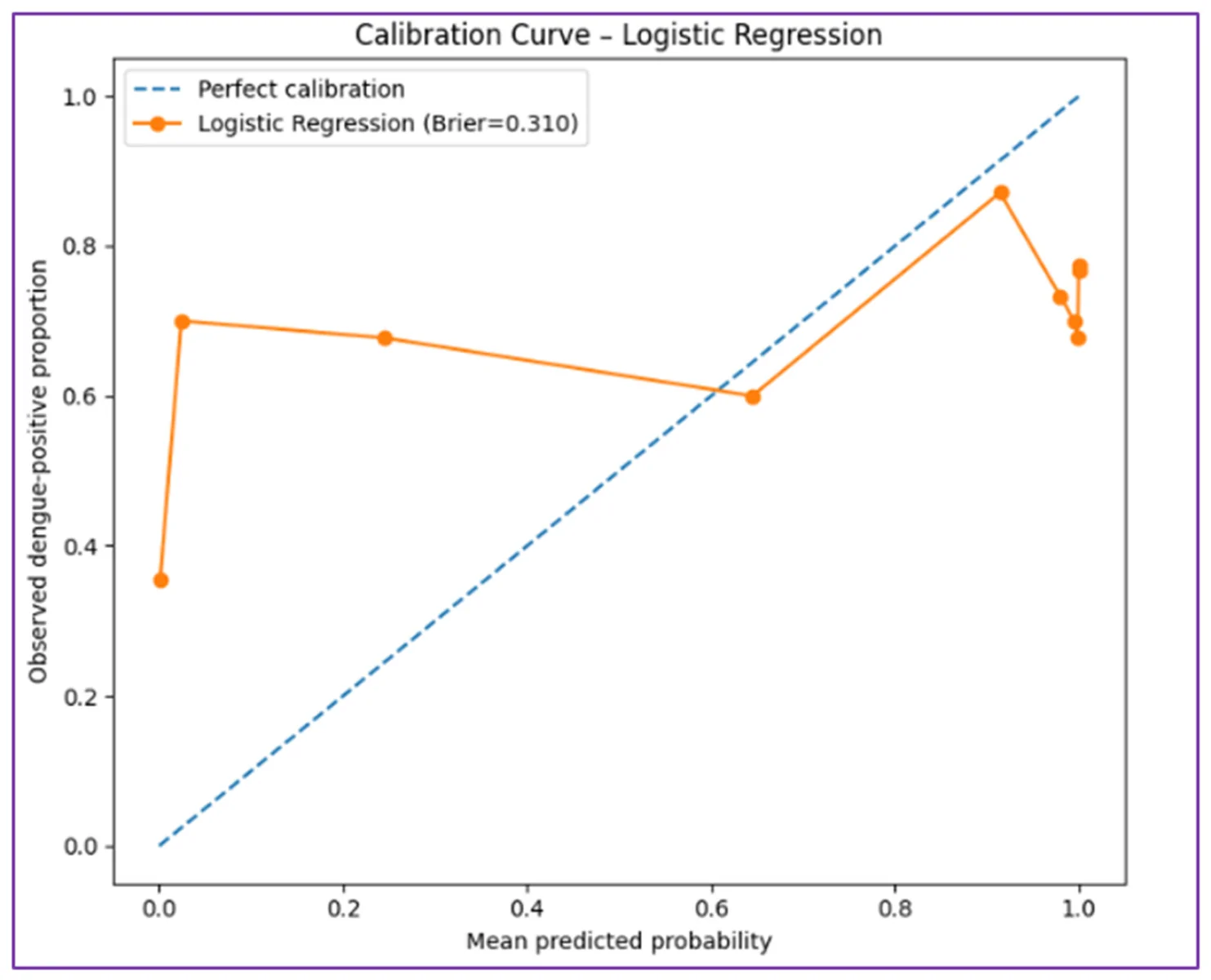
Calibration curve of the L2-penalized LR model.

Figure 6 shows that the calibration curve of the LR model on the held-out test set demonstrated deviations from the line of perfect calibration across several predicted-probability ranges. Consistent with this pattern, the LR Brier score (0.310) was higher than those obtained for RF (0.176), XGBoost (0.175), and LightGBM (0.174) (Table 1). These findings indicate that, in the primary SMOTE-based analysis using the same predictor set and evaluation framework, LR showed numerically lower discrimination and a higher Brier score than the tree-based models.

#### Sensitivity Analysis of LR Without SMOTE

Because SMOTE alters the class distribution of the training data and may consequently affect predicted probabilities, calibration, and probability-based performance measures, an additional sensitivity analysis was conducted by refitting the L2-penalized LR model without SMOTE. To isolate the effect of oversampling, all other analytical settings were kept unchanged, including the same 18 predictors, stratified 80/20 training–test split, standardization procedure, L2 regularization (C = 1.0), five-fold stratified cross-validation for training-derived threshold selection, and the same held-out test set. The model was therefore trained using the original class distribution of the training data.

Table 4 shows that, at the default probability threshold of 0.50, LR without SMOTE achieved an AUROC of 0.655 (95% CI: 0.585–0.724) and an AUPRC of 0.779 (95% CI: 0.719–0.844). Accuracy was 0.734, sensitivity was 0.957 (95% CI: 0.927–0.981), and specificity was 0.250 (95% CI: 0.165–0.344). The corresponding PPV, NPV, and F1-score were 0.735, 0.727, and 0.832, respectively. The Brier score was 0.194 (95% CI: 0.171–0.217). The training-derived threshold maximizing Youden’s J statistic was 0.614. At this threshold, accuracy was 0.705, sensitivity was 0.847, specificity was 0.396, PPV was 0.753, NPV was 0.543, and the F1-score was 0.797. Thus, increasing the threshold reduced sensitivity while improving specificity relative to the default 0.50 threshold.

Table 5 shows that, to specifically examine the influence of SMOTE on LR, the results of the sensitivity analysis were compared with those of the primary SMOTE-trained L2-penalized LR model. Removal of SMOTE increased the AUROC from 0.608 to 0.655 and the AUPRC from 0.753 to 0.779. Most notably, the Brier score decreased from 0.310 to 0.194.

**Figure 7:**
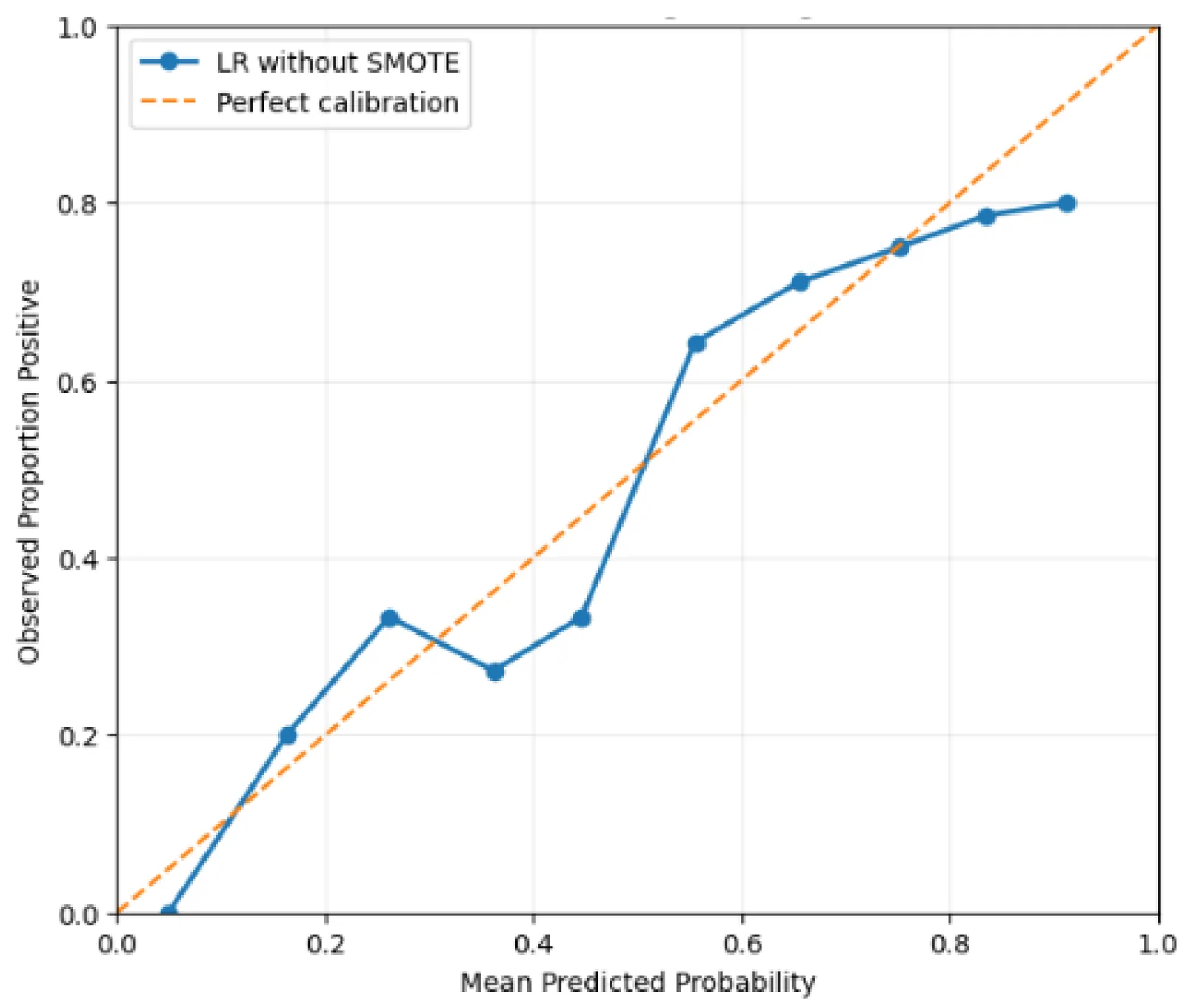
Calibration curve of the L2-penalized LR model without SMOTE on the held-out test set.

Figure 7 shows that the predicted probabilities from LR without SMOTE generally tracked the observed event frequencies, although deviations from the line of perfect calibration remained, particularly in some intermediate and higher probability ranges. Consistent with this pattern, the Brier score decreased substantially from 0.310 in the SMOTE-trained model to 0.194 when the model was trained using the original class distribution.

Overall, these findings indicate that the probability-based performance of LR was sensitive to the use of SMOTE. In particular, the substantial reduction in the Brier score after removal of SMOTE suggests that the higher Brier score observed for the primary SMOTE-trained LR model was at least partly associated with the altered training-set class distribution introduced by oversampling. Accordingly, comparisons of Brier scores between the SMOTE-trained LR and tree-based models should not be interpreted solely as reflecting inherent differences between model classes.

### 3.6. Model Explainability Results

#### 3.6.1. SHAP Analysis

**Figure 8:**
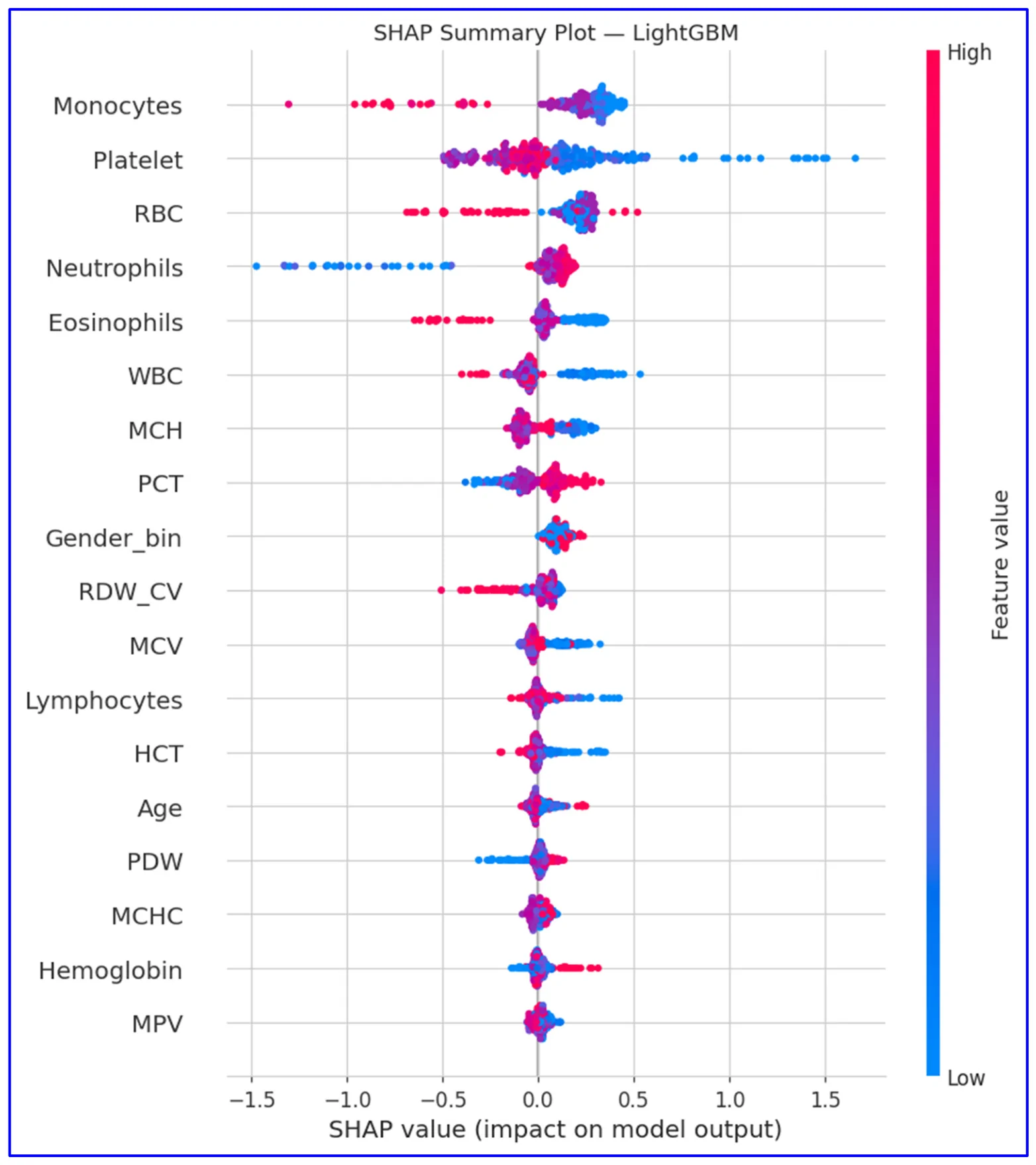
Global SHAP summary plot showing feature contributions to LightGBM predictions. Features are ordered according to their overall SHAP importance. Each point represents one observation, and color indicates the corresponding feature value from low to high. Positive SHAP values shift the model output toward dengue-positive classification, whereas negative values shift the output toward dengue-negative classification.

Figure 8 shows that monocyte percentage was identified as the most influential feature contributing to model predictions. Lower monocyte values tended to shift model output toward the positive class, whereas higher monocyte values shifted predictions toward the negative class. Platelet count ranked second in importance, and lower platelet values tended to shift model predictions toward the dengue-positive class.

#### 3.6.2. Permutation Importance Analysis

**Figure 9:**
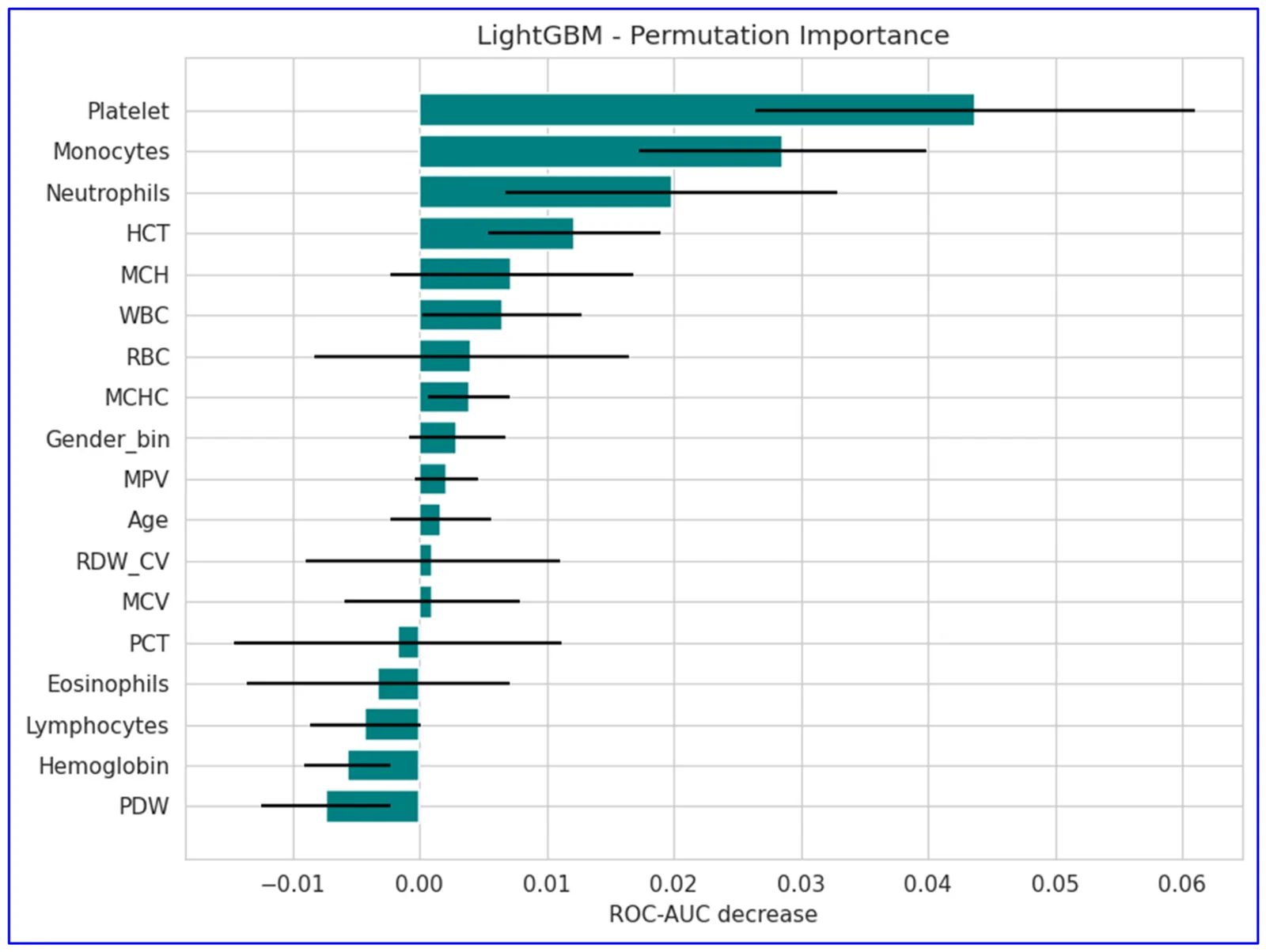
Permutation-based feature importance for the LightGBM model. Bars represent the mean decrease in ROC-AUC after random permutation of each feature, and error bars represent the standard deviation across 20 permutation repetitions. Features are ordered from the largest to the smallest mean decrease in ROC-AUC. Negative values indicate that permutation of the corresponding feature did not reduce model performance and may reflect sampling variability.

Table 6 and Figure 9 show that random shuffling of platelet count produced the largest reduction in model performance (mean decrease in ROC-AUC = 0.0437 ± 0.0172), followed by monocyte percentage (0.0286 ± 0.0113) and neutrophil percentage (0.0198 ± 0.0130). Hematocrit and MCH ranked next, with smaller contributions. Together with the SHAP analysis, these findings consistently identified platelet count and monocyte percentage among the most influential variables in the LightGBM model, while permutation importance additionally ranked neutrophil percentage among the leading predictors.

#### 3.6.3. Stability of Feature-Importance Findings Across Models

Table 7 shows that, to assess whether the feature-importance findings were consistent across algorithms, permutation-importance rankings were compared across RF, XGBoost, and LightGBM. Feature rankings showed significant positive correlations between RF and XGBoost (Spearman’s ρ = 0.790, *p* < 0.001), RF and LightGBM (ρ = 0.600, *p* = 0.009), and XGBoost and LightGBM (ρ = 0.917, *p* < 0.001). Notably, platelet count, monocyte percentage, and neutrophil percentage were ranked first, second, and third, respectively, in all three models. The top-five feature sets showed 60% overlap between RF and XGBoost, 60% between RF and LightGBM, and 80% between XGBoost and LightGBM. Overall, these findings indicate that the principal feature-importance patterns were reasonably stable across algorithms, although some variation was observed among lower-ranked predictors.

### 3.7. Sensitivity Analysis: Effect of SMOTE

To evaluate whether synthetic oversampling materially influenced model performance, a sensitivity analysis was conducted by repeating the complete model-development and evaluation workflow without SMOTE. The same stratified 80/20 training–test split, Optuna-based hyperparameter optimization with five-fold stratified cross-validation, and untouched held-out test set were retained. Thus, the presence or absence of SMOTE was the principal difference between the two analytical pipelines.

Table 8 shows that, at the default classification threshold of 0.50, omission of SMOTE produced only modest changes in overall discrimination. For RF, the AUROC was 0.701 (95% CI: 0.630–0.766) without SMOTE compared with 0.704 (95% CI: 0.632–0.771) with SMOTE. For LightGBM, the corresponding AUROCs were 0.676 (95% CI: 0.603–0.747) and 0.709 (95% CI: 0.636–0.776), while XGBoost yielded AUROCs of 0.675 (95% CI: 0.605–0.740) and 0.702 (95% CI: 0.634–0.766), respectively. AUPRC values were also broadly similar between the two analytical strategies.

Differences were more apparent in the sensitivity–specificity operating profiles. Without SMOTE, sensitivity remained high for all three models, ranging from 0.952 to 0.957, whereas specificity ranged from 0.344 to 0.385. With SMOTE, sensitivity ranged from 0.923 to 0.962 and specificity from 0.385 to 0.438. Importantly, the SMOTE-based pipeline yielded higher AUROC values for all three models, with the largest increases observed for LightGBM (0.676 to 0.709) and XGBoost (0.675 to 0.702), while RF remained essentially unchanged (0.701 to 0.704). SMOTE was also associated with higher specificity for RF (0.344 to 0.438) and XGBoost (0.375 to 0.417), whereas LightGBM specificity remained unchanged at 0.385. These findings indicate that the SMOTE-based pipeline showed modest improvements in discrimination and, for two models, specificity, rather than producing a uniform improvement across every performance measure.

Importantly, these gains were not accompanied by a meaningful deterioration in overall probability accuracy. Brier scores without versus with SMOTE were 0.176 versus 0.176 for RF, 0.178 versus 0.174 for LightGBM, and 0.175 versus 0.175 for XGBoost. Thus, within the present dataset and analytical framework, the SMOTE-based pipeline generally preserved probability accuracy while providing modest improvements in discrimination and/or specificity, supporting its use in the primary analysis. Nevertheless, because oversampling alters the class distribution encountered during model fitting, these findings do not establish that SMOTE is universally beneficial or that it has no effect on probability calibration. Accordingly, calibration findings from the SMOTE-based models should still be interpreted cautiously, and independent validation would be required to determine whether these effects are reproducible in other populations.

## 4. Discussion

In the present study, RF, XGBoost, and LightGBM showed closely similar discriminatory performance for dengue classification, with LightGBM yielding the numerically highest AUROC (0.709), followed by RF (0.704) and XGBoost (0.702). Although LightGBM also achieved the highest sensitivity at the default threshold, its specificity remained limited, and the small absolute differences in AUROC did not indicate a clear discriminatory advantage of one tree-based algorithm over the others. The convergence of performance across the three tree-based algorithms may also reflect the characteristics of the predictor space rather than the equivalence of the algorithms themselves. The present analysis was restricted to 18 routinely available demographic and hematological variables, representing a relatively compact and clinically homogeneous feature set. Under these conditions, RF, XGBoost, and LightGBM may capture largely overlapping predictive information despite differences in their ensemble architectures. Thus, increasing algorithmic complexity alone may provide limited incremental discrimination when the available predictor information is constrained. This interpretation is also supported by the cross-model permutation-importance analysis performed for all three algorithms, which demonstrated substantial agreement in feature rankings, with platelet count, monocyte percentage, and neutrophil percentage consistently ranking among the leading predictors across RF, XGBoost, and LightGBM.

This pattern is broadly consistent with Jahan et al. [21], who analyzed the same 1523-patient Bangladesh dataset using a Tree-Augmented Naive Bayes framework and reported high sensitivity but limited specificity. In contrast, Tuhin et al. [22] reported substantially higher classification performance using the same source dataset with the DengueStackX-19 ensemble framework. Important methodological differences, however, limit direct comparison of these performance estimates. Jahan et al. [21] evaluated models using 10-fold cross-validation and discretized continuous hematological variables, while their evaluation focused primarily on discrimination and classification metrics without calibration or decision-analytic assessment. In contrast, Tuhin et al. evaluated a substantially more complex stacking architecture together with multiple resampling strategies; notably, their reported workflow describes class balancing during preprocessing before the subsequent 80/20 data partition and does not clearly state that synthetic resampling was restricted to training folds, which may introduce a risk of information leakage and optimistic performance estimation. In the present study, a deliberately conservative, leakage-conscious development strategy was adopted. The stratified 80/20 train–test split was performed before model development, and the held-out test set remained completely isolated from resampling, hyperparameter optimization, and threshold selection. SMOTE was applied exclusively within the training workflow and, during cross-validation, only to the training portion of each fold. Hyperparameter optimization with Optuna was conducted using stratified five-fold cross-validation within the training set, without access to the held-out test data, and the optimized classification threshold was derived from out-of-fold training predictions rather than from the test set. The final test set was therefore reserved exclusively for final performance evaluation. Consequently, differences in reported performance across studies using the same source data may reflect differences in preprocessing, resampling, validation design, hyperparameter optimization, threshold selection, and model complexity rather than algorithm choice alone. Within the present framework, the combination of moderate discrimination, high sensitivity, and limited specificity supports a potential screening or triage-support role rather than standalone diagnostic use.

The inclusion of LR provided a conventional statistical benchmark for interpreting the performance of the tree-based models. Using the same predictor set and held-out test set, the primary SMOTE-trained LR yielded a numerically lower AUROC than LightGBM, RF, and XGBoost (0.608 vs. 0.709, 0.704, and 0.702, respectively) and a higher Brier score (0.310 vs. 0.174, 0.176, and 0.175, respectively). These findings suggest that the tree-based models may have captured nonlinear relationships or interactions that were not represented by the main-effects LR model. However, the comparison was based on a single internally partitioned dataset, and no formal statistical comparison of the AUROCs was performed. The observed differences should therefore not be interpreted as evidence of the general superiority of tree-based algorithms over LR. Rather, they indicate that, under the predictor set and analytical framework used in the present study, the tree-based models showed numerically better discrimination and lower Brier scores than the primary SMOTE-trained LR. External validation is required to determine whether these differences are reproducible in independent populations.

A second finding was that none of the three evaluated tree-based algorithms demonstrated a meaningful advantage in discrimination despite their different learning architectures. This observation is consistent with the broader dengue ML literature, in which the reported best-performing algorithm varies markedly across datasets, populations, and outcomes. A summary of artificial intelligence-based studies published between 2016 and 2026 that used demographic, clinical, and biochemical data for dengue-related prediction, prognosis, and diagnosis is provided in Table 9 [17,18,19,20,21,22,27,28,29,30,31,32,33,34,35,36,37,38,39,40,41,42,43,44,45,46,47,48,49,50,51,52,53,54,55,56,57,58,59]. RF was selected as the leading model in some cohorts, with Nguyen et al. [33] reporting an AUC of 0.945 and accuracy of 0.951 and Thanh et al. [37] reporting an AUC of 0.970 and accuracy of 0.910. In contrast, SVM-based approaches were favored in other studies, including Qaiser et al. [42] and Ojeda Meixueiro et al. [34], although their reported performance varied considerably; Ojeda Meixueiro et al. [34] reported an AUC of 0.713 and accuracy of 0.680, whereas Saleem et al. [48] reported an accuracy of 0.995 in a small blinded dataset. Boosted and ensemble approaches also performed strongly in several studies, including AUC 0.991 with Extra Trees [51], AUC 0.992 with stacking [41], and AUCs of 0.947–0.977 in the two studies by Madewell et al. [31,32]; Riya et al. [41] likewise reported strong performance with an ensemble-based approach. Lu et al. [20] further demonstrated that LR remained competitive across multiple datasets, with external-validation AUCs ranging from 0.55 to 0.89, underscoring that apparent superiority may be dataset-dependent rather than algorithm-specific. This variability is also emphasized by the systematic reviews of Andrade Girón et al. [9] and Hoyos et al. [10], which documented substantial heterogeneity in algorithms, predictor sets, outcome definitions, case definitions, local prevalence, and validation strategies across dengue ML studies. Accordingly, the closely similar AUROCs observed in the present study suggest that, within this dataset and predictor set, discrimination was not strongly dependent on the choice among these three tree-based algorithms. The slight numerical advantage of LightGBM should therefore not be overinterpreted as evidence of intrinsic algorithmic superiority; rather, the findings suggest that, within this hematology-only dataset, the evaluated tree-based methods relied on broadly similar predictive information.

The present study also demonstrated that model operating characteristics changed materially when the decision threshold was optimized. For LightGBM, increasing the threshold from 0.50 to 0.558 improved specificity from 0.385 to 0.458 while sensitivity decreased from 0.962 to 0.914. A comparable high-sensitivity/low-specificity pattern was reported by Jahan et al. [21] using the same hematological dataset, whereas the wider literature summarized in Table 9 shows considerable variation in sensitivity and specificity across diagnostic models. Such differences may partly reflect variation in class prevalence, predictor composition, validation procedures, and the classification thresholds used to convert predicted probabilities into binary outcomes. Importantly, the present threshold optimization changed the balance between false-positive and false-negative classifications but did not alter AUROC or the intrinsic ranking ability of the model. Thus, threshold selection should be regarded as determination of an operating point according to the intended use rather than as an improvement in model discrimination. The persistence of relatively modest specificity despite threshold optimization further suggests that threshold adjustment alone cannot overcome limitations in the discriminatory information contained in routine hematological predictors.

Another important finding was that the evaluation of LightGBM extended beyond discrimination to probability calibration and decision-analytic performance. The calibration curve showed closer agreement between predicted and observed probabilities in the low-to-moderate probability range, with greater deviation at higher predicted probabilities, and the model yielded a Brier score of 0.174. These findings should be interpreted in the context of the class-imbalance strategy used during model development. Although SMOTE was restricted to the training data and the held-out test set retained the original class distribution, oversampling altered the class distribution encountered during model fitting and may consequently have affected the scale of the predicted probabilities. Because no post-hoc probability correction or recalibration was performed, the observed departures from ideal calibration may partly reflect probability distortion associated with oversampling. This consideration also applies to probability-dependent assessments such as the Brier score and decision curve analysis. The reported findings therefore represent the performance of the uncorrected probabilities generated by the SMOTE-trained model and should not be interpreted as evidence of fully calibrated risk estimates.

DCA suggested potential net benefit across a range of threshold probabilities; however, the evaluated range was not prespecified as clinically optimal and the analysis did not establish clinical utility. The observed high-sensitivity, low-specificity profile was more consistent with a potential adjunctive screening or triage function than with confirmatory diagnosis. In settings where immediate etiological testing is unavailable, such a model might help identify patients who warrant prioritized confirmatory testing, closer assessment, or follow-up. Lower decision thresholds may be relevant when minimizing missed dengue cases is the primary objective, whereas higher thresholds may reduce false-positive referrals when downstream testing capacity is limited. Nevertheless, the model’s limited specificity could result in a substantial false-positive burden and increased clinical workload. It should therefore not replace NS1 antigen testing, RT-PCR, or other established diagnostic procedures or be used as a standalone diagnostic rule. These findings complement the cross-dataset calibration differences reported by Lu et al. [20] and the Brier-score assessment and external validation undertaken by Sangkaew et al. [18]. Other studies, including Velasco et al. [19] and Nguyen et al. [33], similarly illustrate the increasing emphasis on evaluating prediction models beyond discrimination alone. By jointly assessing discrimination, calibration, threshold-dependent performance, and decision-analytic net benefit, the present study provides a broader characterization of model performance; however, geographically independent and prospective validation remains necessary before clinical utility can be established.

The explainability analyses identified platelet count and monocyte percentage as influential variables across both SHAP and permutation-importance approaches, while neutrophil percentage was additionally prominent in permutation importance. These findings are biologically plausible in the context of dengue pathophysiology. Alterations in platelet dynamics during dengue may reflect interacting mechanisms including impaired platelet production, peripheral destruction, immune-mediated effects, and consumption. Changes in monocyte and neutrophil proportions may likewise reflect the evolving innate immune response to infection, with monocytes representing important cellular targets involved in viral replication and immune activation. The consistent prominence of these variables across complementary explainability approaches therefore supports the biological plausibility of the predictive patterns identified, although their importance should be interpreted as model-dependent predictive contribution rather than evidence of causal effects or clinically validated laboratory thresholds. A particularly informative finding was the behavior of platelet count. Importantly, the SHAP results should not be interpreted as establishing clinically validated laboratory cut-offs. They describe model-dependent patterns of contribution across the observed feature distributions rather than causal or diagnostic thresholds. Derivation of clinically actionable cut-off values for platelet count, monocyte percentage, or neutrophil percentage would require dedicated threshold analyses followed by prospective external validation. In the conventional univariable analysis reported by Nirob et al. [23], platelet count did not differ significantly between dengue-positive and dengue-negative groups (*p* = 0.1621); nevertheless, in the present ML analysis it emerged as the leading variable in permutation importance and among the most influential features in SHAP analysis. This contrast highlights an important methodological distinction: conventional univariable comparisons evaluate variables in isolation, whereas ML models can exploit multivariable, nonlinear, and interaction-dependent predictive information. Therefore, the absence of a statistically significant univariable difference does not preclude a variable from contributing meaningfully to classification within a multivariable model. Using the same source data, Tuhin et al. [22] also identified platelet count, monocytes, hematocrit, neutrophils, and RBC-related indices among influential features, while Jahan et al. [21] identified interpretable dependency structures involving platelet count, hematocrit, and leukocyte variables. More broadly, Dasgupta et al. [28] and Riya et al. [41] also demonstrated the predictive value of routine hematological variables in dengue classification using ML-based approaches. The repeated identification of platelet and leukocyte-related variables across different ML frameworks therefore supports their predictive relevance while illustrating how multivariable modeling can reveal information not apparent from isolated group comparisons. Importantly, the feature-importance findings were not confined to the representative LightGBM model. In the cross-model stability analysis, permutation-importance rankings showed moderate-to-strong positive agreement across the three algorithms, with Spearman’s ρ values ranging from 0.600 to 0.917. The strongest agreement was observed between XGBoost and LightGBM (ρ = 0.917), while RF also showed substantial agreement with XGBoost (ρ = 0.790) and moderate agreement with LightGBM (ρ = 0.600). More importantly, platelet count, monocyte percentage, and neutrophil percentage occupied the first, second, and third positions, respectively, in the permutation-importance rankings of all three models. The overlap among the five highest-ranked predictors was 60% between RF and XGBoost, 60% between RF and LightGBM, and 80% between XGBoost and LightGBM. This cross-model consistency strengthens the interpretation that the prominence of the leading hematological predictors was not specific to LightGBM alone, although some model-dependent variability remained among the lower-ranked features. However, these feature-importance rankings should not be interpreted as a definitive hierarchy of hematological biomarkers. Several CBC-derived variables are biologically and statistically correlated, and both SHAP and permutation importance may distribute importance differently among correlated predictors. Accordingly, the prominence of platelet count, monocyte percentage, and neutrophil percentage should be interpreted as their relative predictive contribution within the fitted models rather than evidence that these variables are independently or biologically more important than all other hematological parameters. Correlations among the included predictors are presented in Supplementary Figure S1 to facilitate interpretation of the explainability findings. These findings nevertheless represent predictive associations rather than causal or etiological effects.

The moderate discrimination observed in the present study should also be interpreted in relation to the clinical scope of the available predictors and outcome. Table 9 demonstrates that apparently high-performing dengue ML models have been developed for substantially different clinical questions, including diagnostic classification, hospitalization, severe dengue, plasma leakage, and dengue shock syndrome. For example, Velasco et al. [19] investigated hospitalization risk, Anh et al. [27] evaluated risk stratification for hospitalization, Jean Pierre et al. [17] examined severity prediction, and Tran et al. [38] and Nguyen et al. [33] addressed dengue shock syndrome. The prognostic information available for these endpoints may include clinical manifestations, biomarkers, disease severity indicators, or temporal information that were unavailable in the present CBC-based dataset. Conversely, diagnosis-oriented studies such as Dasgupta et al. [28], Madewell et al. [31,32], Qaiser et al. [42], Riya et al. [41], and Saleem et al. [48] used different populations, predictor structures, and validation designs. Consequently, numerical performance estimates across these studies are not directly interchangeable. The principal gap revealed by this literature is therefore not simply the need for more complex algorithms, but the need for reproducible validation across clinically comparable populations, predictors, outcomes, and settings. In this context, the moderate AUROC observed in the present study is best interpreted as the performance achievable from the available routine demographic and hematological information under the specified internal validation framework, rather than as evidence of inadequate algorithmic complexity.

The present study incorporated several methodological safeguards that strengthen interpretation of its internal performance estimates. The held-out test set was excluded from hyperparameter optimization, resampling, and threshold selection; SMOTE was confined to the training workflow and to the training component of each cross-validation fold; and threshold optimization was derived from out-of-fold training predictions rather than test-set performance. In addition, the study evaluated AUROC and AUPRC together with threshold-dependent classification metrics, calibration, Brier score, decision curve analysis, SHAP, and permutation importance. These features are particularly relevant given the methodological heterogeneity and concerns regarding model generalizability highlighted by Andrade Girón et al. [9], Hoyos et al. [10], and Lu et al. [20]. Together, these procedures reduce direct information leakage and permit a multidimensional assessment of model behavior. They strengthen the internal evaluation of the present models but should not be interpreted as substitutes for external validation.

### Limitations

The findings should be interpreted in light of several limitations. First, both model development and held-out testing were performed using a single publicly available source dataset from Bangladesh. Although the test set was completely separated from hyperparameter optimization, resampling, and threshold determination, it originated from the same source population as the training data. The study therefore provides internal hold-out validation rather than external validation, and the transportability of discrimination, calibration, operating thresholds, and decision-curve-based net benefit across populations with different dengue prevalence, demographic and clinical characteristics, healthcare settings, laboratory platforms and measurement distributions, or circulating viral serotypes remains uncertain. In addition, final model performance was evaluated using a single stratified 80/20 train–test partition. Although 95% bootstrap confidence intervals based on 2000 resamples of the held-out test set were used to quantify sampling uncertainty around the performance estimates, this approach does not assess the stability of model rankings across alternative train–test partitions. Given the small numerical differences in AUROC among the three algorithms, their relative ranking may therefore be sensitive to the particular data partition used. Repeated resampling across multiple train–test partitions would provide complementary information regarding internal ranking stability and should be considered in future validation studies. This limitation is particularly relevant in view of the cross-dataset variability demonstrated by Lu et al. [20] and the use of independent validation populations in studies such as Sangkaew et al. [18].

Second, the available predictors were restricted to demographic characteristics and routine hematological parameters. Illness day, symptoms, warning signs, NS1 antigen status, RT-PCR results, serotype, comorbidities, treatment information, and longitudinal laboratory changes were unavailable. The models could therefore not incorporate clinical, temporal, or virological information that may provide complementary discriminatory information and may partly explain the limited specificity observed in the present study. Studies addressing hospitalization or severity, including Anh et al. [27], Jean Pierre et al. [17], Velasco et al. [19], Tran et al. [38], and Sangkaew et al. [18], illustrate the additional predictive information that may become available when richer clinical data are incorporated. However, these studies address different outcomes and should not be regarded as direct performance comparators.

Third, routine hematological abnormalities are not specific to dengue. At the optimized LightGBM threshold, 52 false-positive and 18 false-negative classifications remained; however, detailed clinical profiling of these misclassified cases was not possible because the secondary dataset did not include illness duration, comorbidities, detailed symptom profiles, or sufficiently detailed alternative diagnoses among dengue-negative patients. Consequently, it could not be determined whether prediction errors were concentrated among particular competing febrile illnesses, hematological conditions, or stages of disease, and the present analysis cannot establish the model’s ability to distinguish dengue from specific alternative infections such as malaria or chikungunya. Future validation studies using clinically richer datasets should specifically characterize misclassified patients to identify systematic sources of prediction error and guide model refinement.

Fourth, the available source documentation does not characterize the diagnostic reference standard underlying the binary dengue-positive and dengue-negative outcome in sufficient detail to determine precisely how diagnostic status was established for every record. Because the present study was a secondary analysis, the outcome definition could not be independently reassessed. Any misclassification in the source outcome could propagate into both model development and performance evaluation; consequently, this uncertainty should be considered when interpreting the models as diagnostic classifiers.

Finally, this was a retrospective secondary analysis of existing anonymized data. The findings therefore describe predictive relationships within the available dataset and cannot establish causality, prospective clinical effectiveness, or improvement in patient outcomes. Similarly, feature importance derived from SHAP or permutation analysis should not be interpreted as evidence that the identified hematological variables cause dengue positivity or determine its biological course. External validation should be prioritized in independent dengue-endemic populations, initially in settings with comparable resource constraints and routine laboratory availability, followed by evaluation across regions with different epidemiological and healthcare contexts. Future datasets should also incorporate clinically informative variables unavailable in the present dataset, particularly duration of illness, symptom profiles, comorbidities, and etiological test results. Such information would permit assessment of disease-stage-specific performance, more detailed characterization of misclassification, and evaluation of whether readily obtainable clinical information provides incremental value beyond routine hematological parameters alone. Prospective evaluation will ultimately be required before clinical implementation or transportability can be established.

## 5. Conclusions

Tree-based machine-learning models based on routine demographic and hematological parameters provided moderate discrimination, with relatively high sensitivity but limited specificity, for dengue classification. LightGBM yielded the numerically highest AUROC; however, the small differences among LightGBM, RF, and XGBoost did not support the superiority of any individual tree-based algorithm. Under the same predictor set and internal evaluation framework, the tree-based models yielded numerically higher AUROCs and lower Brier scores than the LR comparator. Nevertheless, these findings were derived from a single internally partitioned dataset and should not be interpreted as establishing the general superiority of tree-based methods over LR. Evaluation of discrimination, threshold-dependent classification, calibration, potential decision-analytic net benefit, and complementary explainability measures demonstrated that model performance cannot be adequately characterized by AUROC or accuracy alone. The findings support the potential use of CBC-based machine learning as an adjunctive screening or triage-support approach rather than as a standalone diagnostic tool. External and prospective validation in independent populations, clearer characterization of the diagnostic reference standard, and integration of hematological predictors with clinical and confirmatory diagnostic information are required before the transportability and clinical usefulness of these models can be established.

## Figures and Tables

**Table 1:** Test set performance metrics of the three models using the default threshold of 0.5.

Metric	LightGBM	RF	XGBoost
AUROC	0.709 (0.636–0.776)	0.704 (0.632–0.771)	0.702 (0.634–0.766)
AUPRC	0.785 (0.723–0.854)	0.796 (0.731–0.861)	0.792 (0.729–0.856)
Accuracy	0.780	0.771	0.780
Sensitivity	0.962 (0.932–0.986)	0.923 (0.887–0.957)	0.947 (0.914–0.976)
Specificity	0.385 (0.288–0.485)	0.438 (0.337–0.533)	0.417 (0.320–0.516)
PPV	0.773	0.781	0.780
NPV	0.822	0.724	0.784
F1-score	0.857	0.847	0.855
Brier score	0.174 (0.155–0.194)	0.176 (0.158–0.196)	0.175 (0.156–0.194)

AUROC, area under the receiver operating characteristic curve; AUPRC, area under the precision–recall curve; LightGBM, Light Gradient Boosting Machine; RF, Random Forest; XGBoost, Extreme Gradient Boosting; PPV, positive predictive value; NPV, negative predictive value; F1-score, harmonic mean of precision and recall.

**Table 2:** Test set performance metrics of the three models using thresholds optimized by Youden’s J statistic.

Metric	LightGBM	RF	XGBoost
Optimized threshold	0.558	0.545	0.537
AUROC	0.709 (0.636–0.776)	0.704 (0.632–0.771)	0.702 (0.634–0.766)
AUPRC	0.785 (0.723–0.854)	0.796 (0.731–0.861)	0.792 (0.729–0.856)
Accuracy	0.771	0.761	0.771
Sensitivity	0.914 (0.874–0.950)	0.895 (0.853–0.934)	0.923 (0.885–0.958)
Specificity	0.458 (0.357–0.560)	0.469 (0.364–0.568)	0.438 (0.337–0.539)
PPV	0.786	0.786	0.781
NPV	0.710	0.672	0.724
F1-score	0.845	0.837	0.847
Brier score	0.174 (0.155–0.194)	0.176 (0.158–0.196)	0.175 (0.156–0.194)

AUROC, area under the receiver operating characteristic curve; AUPRC, area under the precision–recall curve; LightGBM, Light Gradient Boosting Machine; RF, Random Forest; XGBoost, Extreme Gradient Boosting; PPV, positive predictive value; NPV, negative predictive value; F1-score, harmonic mean of precision and recall.

**Table 3:** Performance of the L2-penalized LR model on the held-out test set.

Performance Metric	Default Threshold (0.500)	Optimal Threshold Youden’s J (0.108)
AUROC (95% CI)	0.608 (0.536–0.677)	0.608 (0.536–0.677)
AUPRC (95% CI)	0.753 (0.688–0.820)	0.753 (0.688–0.820)
Accuracy	0.646	0.669
Sensitivity (95% CI)	0.742 (0.682–0.800)	0.837 (0.787–0.885)
Specificity (95% CI)	0.438 (0.337–0.537)	0.302 (0.213–0.394)
PPV	0.742	0.723
NPV	0.438	0.460
F1-score	0.742	0.776
Brier score (95% CI)	0.310 (0.266–0.356)	0.310 (0.266–0.356)

CI, confidence interval; AUROC, area under the receiver operating characteristic curve; AUPRC, area under the precision–recall curve; PPV, positive predictive value; NPV, negative predictive value.

**Table 4:** Performance of L2-penalized LR without SMOTE on the held-out test set.

Performance Metric	Default Threshold (0.50)	Training-Derived Threshold (0.614)
AUROC (95% CI)	0.655 (0.585–0.724)	0.655 (0.585–0.724)
AUPRC (95% CI)	0.779 (0.719–0.844)	0.779 (0.719–0.844)
Accuracy	0.734 (0.685–0.784)	0.705 (0.652–0.757)
Sensitivity (95% CI)	0.957 (0.927–0.981)	0.847 (0.796–0.895)
Specificity (95% CI)	0.250 (0.165–0.344)	0.396 (0.296–0.495)
PPV	0.735 (0.682–0.788)	0.753 (0.698–0.808)
NPV	0.727 (0.559–0.880)	0.543 (0.422–0.662)
F1-score	0.832 (0.795–0.866)	0.797 (0.755–0.838)
Brier score (95% CI)	0.194 (0.171–0.217)	0.194 (0.171–0.217)

**Table 5:** Sensitivity analysis comparing L2-penalized LR with and without SMOTE.

Performance Metric	LR with SMOTE	LR Without SMOTE	Absolute Difference
AUROC	0.608 (0.536–0.677)	0.655 (0.585–0.724)	+0.047
AUPRC	0.753 (0.688–0.820)	0.779 (0.719–0.844)	+0.026
Accuracy	0.646	0.734	+0.088
Sensitivity	0.742 (0.682–0.800)	0.957 (0.927–0.981)	+0.215
Specificity	0.438 (0.337–0.537)	0.250 (0.165–0.344)	−0.188
PPV	0.742	0.735	−0.007
NPV	0.438	0.727	+0.289
F1-score	0.742	0.832	+0.090
Brier score	0.310 (0.266–0.356)	0.194 (0.171–0.217)	−0.116

**Table 6:** Permutation importance analysis results for the LightGBM model.

Rank	Variable	Mean Importance	SD of Importance
1	Platelet	0.0437	0.0172
2	Monocyte	0.0286	0.0113
3	Neutrophil	0.0198	0.0130
4	Hematocrit	0.0121	0.0068
5	MCH	0.0073	0.0095
6	WBC	0.0065	0.0063
7	RBC	0.0041	0.0124
8	MCHC	0.0039	0.0032
9	Sex	0.0029	0.0038
10	MPV	0.0021	0.0025

SD, standard deviation; MCH, mean corpuscular hemoglobin; WBC, white blood cell count; RBC, red blood cell count; MCHC, mean corpuscular hemoglobin concentration; MPV, mean platelet volume.

**Table 7:** Stability of permutation-based feature-importance rankings across the three machine-learning models.

Metric	RF vs. XGBoost	RF vs. LightGBM	XGBoost vs. LightGBM
Spearman’s ρ	0.790	0.600	0.917
*p*-value	<0.001	0.009	<0.001
Common Top-5 Features	Platelet count; Monocyte percentage; Neutrophil percentage	Platelet count; Monocyte percentage; Neutrophil percentage	Platelet count; Monocyte percentage; Neutrophil percentage; MCH
Common features, n/5	3/5	3/5	4/5
Top-5 overlap (%)	60	60	80

MCH, mean corpuscular hemoglobin. ρ, Spearman’s rank correlation coefficient. Spearman’s ρ was calculated using permutation-importance rankings across all 18 predictors. Platelet count, monocyte percentage, and neutrophil percentage were consistently ranked first, second, and third, respectively, in all three models. Top-5 overlap represents the proportion of the five highest-ranked predictors shared between each pair of models.

**Table 8:** Sensitivity analysis comparing model performance with and without SMOTE at the default classification threshold of 0.50.

Metric	RF-SMOTE	RF-No SMOTE	LightGBM- SMOTE	LightGBM- No SMOTE	XGBoost-SMOTE	XGBoost-No SMOTE
AUROC	0.704 (0.632–0.771)	0.701 (0.630–0.766)	0.709 (0.636–0.776)	0.676 (0.603–0.747)	0.702 (0.634–0.766)	0.675 (0.605–0.740)
AUPRC	0.796 (0.731–0.861)	0.793 (0.726–0.860)	0.785 (0.723–0.854)	0.771 (0.709–0.840)	0.792 (0.729–0.856)	0.770 (0.703–0.840)
Accuracy	0.771	0.761	0.780	0.777	0.780	0.774
Sensitivity	0.923 (0.887–0.957)	0.952 (0.922–0.979)	0.962 (0.932–0.981)	0.957 (0.926–0.981)	0.947 (0.914–0.976)	0.957 (0.928–0.981)
Specificity	0.438 (0.337–0.533)	0.344 (0.248–0.438)	0.385 (0.288–0.485)	0.385 (0.286–0.484)	0.417 (0.320–0.516)	0.375 (0.280–0.469)
PPV	0.781	0.760	0.773	0.772	0.780	0.769
NPV	0.724	0.767	0.822	0.804	0.784	0.800
F1-score	0.847	0.845	0.857	0.855	0.855	0.853
Brier score	0.176 (0.158–0.196)	0.176 (0.154–0.201)	0.174 (0.155–0.194)	0.178 (0.152–0.200)	0.175 (0.156–0.194)	0.175 (0.151–0.200)

SMOTE, Synthetic Minority Over-sampling Technique; AUROC, area under the receiver operating characteristic curve; AUPRC, area under the precision–recall curve; LightGBM, Light Gradient Boosting Machine; RF, Random Forest; XGBoost, Extreme Gradient Boosting; PPV, positive predictive value; NPV, negative predictive value; F1-score, harmonic mean of precision and recall.

**Table 9:** Methodological characteristics and predictive performance of selected machine-learning studies on dengue-related diagnosis, prognosis, and risk prediction.

Author	Years	Country	Sample	Train Set	Test Set	Cross- Validation	Algorithms Used	Chosen Algorithm	Key Results
Sangkaew [18]	2026	Vietnam/Thailand	2769	2245	524	10-fold	LR-WHO warning signs, Lasso-LR, RF, XGBoost, SVM, ANN	Lasso-LR (external validation)	DSS AUC 0.677 Brier 0.059 Combined endpoint AUC 0.624 Brier 0.299
Jahan [21]	2026	Bangladesh	1523	NR	NR	10-fold	TAN, NB, SVM, LR, LDA, KNN	TAN (internal validation)	AUC 0.671 ACC 0.756 Sens 0.961 Spec 0.314
Velasco [19]	2025	Colombia	8814	70%	30%	Monte Carlo CV, 1000 iterations	LR, RF, SVM, Cox model	RF (test validation)	ACC 0.882 Sens 0.872 Spec 0.887 PPV 0.804 F1-score 0.837
Tuhin [22]	2025	Bangladesh	1372 (1523)	80%	20%	10-fold; 5-fold stacking	LR, DT, RF, SVM, LightGBM, KNN, DengueStackX-19	DengueStackX-19 (test validation)	ACC 0.964 Precision 0.942 Sens 0.942 F1-score 0.942
Thanh [37]	2025	Vietnam	524	80%	20%	5-fold	LR, RF, SVM, KNN, NB, AdaBoost, XGBoost	RF (test validation)	AUC 0.970 ACC 0.910 Sens 0.780 Spec 0.960 Precision 0.880 F1-score 0.820
Ojeda Meix [34]	2025	Mexico	1284	90%	10%	10-fold	Efficient linear SVM, Coarse KNN, Optimizable ensemble	Efficient linear SVM (test validation)	ACC 0.680 Precision 0.622 Sens 0.908 F1-score 0.738 AUC 0.713 Kappa 0.359
Nguyen [33]	2025	Vietnam	230	173	57	10-fold CV × 3 repeats	RF, SVM, DT, ANN, NB, KNN	RF (test validation)	AUC 0.945 ACC 0.951 Sens 0.894 Spec 0.976 PPV 0.944 NPV 0.954 Kappa 0.884
Madewell [31]	2025	Puerto Rico	49,679	70%	30%	NR	LR, RF, SVM, ANN, AdaBoost, LightGBM, XGBoost	XGBoost (test validation)	AUC 0.947 ACC 0.879 Sens 0.862 Spec 0.896 Precision 0.893 F1-score 0.877
Madewell [32]	2025	Puerto Rico	1708	70%	30%	5-fold	LR, DT, KNN, NB, SVM, ANN, AdaBoost, CatBoost, LightGBM, XGBoost, ensemble	Ensemble model (test validation)	AUC 0.977 Sens 0.956 Spec 0.933 F1-score 0.945 Kappa 0.889
Lu [20]	2025	Public datasets (Secondary analysis)	9386	Dataset-specific	Other external datasets	Site-level/10-fold CV; cross-dataset external testing	LR, CART/DT, SVM	LR	External validation AUC 0.55–0.89 Internal validation AUC 0.74–0.89
Katz [30]	2025	Vietnam	218 (377)	146	72	5-fold	glmnet, XGBoost	Glmnet (test validation)	AUC 0.980 Sens 0.917 Spec 1.000
Haque [29]	2025	Bangladesh	989 (1003)	791	198	10-fold CV (stratified)	SVC, KNN, DT, ANN	Optimized DT (test validation)	ACC 0.995 AUC 0.996
R [35]	2025	Public datasets (Secondary analysis)	177	NR	NR	5-fold	DT, RF, SVC, SVC-gridCV, LR, RNN, KNN, XGBoost, Extra Trees, DNN, AdaBoost, NB, MLP	Optimized lightweight MLP (internal validation)	AUC 0.970 ACC 0.920 Precision 1.000 Sens 0.830 Spec 1.000 F1-score 0.900
Dasgupta [28]	2025	India	122	98	24	NR	MARS, ANN	ANN (test validation)	ACC 0.958
Anh [27]	2025	Vietnam	299	NR	NR	10-fold	RF	RF biomarker model (internal validation)	AUC 0.860 ACC 0.780 Sens 0.810 Spec 0.770 F1-score 0.810
Tran [38]	2024	Thailand/ Vietnam	4522	3182	1340	NR	LR, RF, XGBoost, SVM	LR nomogram (test validation)	AUC 0.830 ACC 0.820 Sens 0.710 Spec 0.840
Thanh [39]	2024	Vietnam	1278	70%	30%	10-fold	LR, RF, SVM, KNN, XGBoost	SVM (test validation)	AUC 0.963 ACC 0.948 Sens 0.860 Spec 0.959 Precision 0.725 F1-score 0.787
Syfullah [36]	2024	Bangladesh	344 (357)	80%	20%	10-fold	Prophet, LSTM, RF, XGBoost	RF (internal validation)	ACC 0.925 Precision 0.922 Sens 0.937 F1-score 0.914
Ruiz Valdez [40]	2024	Mexico	787	321	466	NR	ANN, direct algorithm, DIRECT-ANN	ANN-ROC (test validation)	AUC 0.899 Sens 0.900 Spec 0.820 PPV 0.810 NPV 0.910 Kappa 0.720
Riya [41]	2024	Bangladesh	320	80%	20%	CV, folds NR	LR, RF, SVM, NB, AdaBoost, XGBoost, LightGBM, MLP, ANN, CNN, GRU, Bi-LSTM, TabPFN, TabTransformer, stacking	Stacking ensemble (test validation)	AUC 0.992 ACC 0.969 Precision 0.977 Sens 0.955 F1-score 0.965
Qaiser [42]	2024	Pakistan	300	70%	30%	NR	LR, XGBoost, LightGBM, RF, SVM, CatBoost	SVM (test validation)	ACC 0.714 Sens 0.974 Precision 0.716
Karolcik [43]	2024	Vietnam	142 (188)	NR	NR	5-fold CV (stratified)	DT, RF, SVM, MLP, CNN	CNN (internal validation)	Precision 0.785 Sens 0.771
Jean Pierre [17]	2024	India	102	NR	NR	5-fold CV	RF, XGBoost, LR, Ridge classifier, SVM, NB	Ridge classifier (internal validation)	AUC 0.920
Chaw [44]	2024	Malaysia	170	119	51	10-fold	LR, DT, SVM, ANN, Bagging, AdaBoost, Gentle AdaBoost, Adaptive LR, Robust Boosting	Bagging (test validation)	AUC 0.790 Precision 0.860 Sens 0.980 F1-score 0.910
Bohm [46]	2024	Brazil	20,000 (364,276)	70%	30%	10-fold	DT, KNN, MLP, LR	MLP (test validation)	AUC 0.988 ACC 0.931 F1-score 0.933
Ansari [45]	2024	India	1148	803	345	5-fold	AdaBoost, Extra Trees, GBDT, KNN, LR, MLP, RF, XGBoost	XGBoost (test validation)	AUC 0.793 ACC 0.872 Sens 0.632 Spec 0.953
Zargari Marandi [47]	2023	Sri Lanka	546 (877)	374	172	2 × 5-fold (Nested)	MDL, RF, LightGBM, average stacking ensemble	DENV5F-AS ensemble (test validation)	AUC 0.800 Sens 0.548 Spec 0.879
Saleem [48]	2022	Pakistan	62	32	30 blind test	10-fold	PCA-DA, PCA-SVM, PLS-DA, PLSR	PCA-SVM Poly-5 (test validation)	ACC 0.995 Sens 0.998 Spec 0.991
Liu [49]	2022	Multicountry/ Colombia	742	365	377	Leave-one-dataset-out CV	XGBoost	XGBoost (external validation)	AUC 0.844 Sens 0.864 Spec 0.797
Hamdani [50]	2022	Indonesia	110	NR	NR	3-, 5-, 10-fold	C4.5, DT, KNN, RF, SVM	SVM (internal validation)	ACC 0.991 Precision 0.991 Sens 0.991
Abdualgalil [51]	2022	Yemen	6501 (6694)	4550	1951	10-fold + holdout	KNN, GBC, ETC, XGBoost, LightGBM	ETC (test validation)	AUC 0.991 ACC 0.991 Sens 0.992 Precision 0.991 F1-score 0.991
Srivastava [52]	2020	India	182	146	36	NR	SVM, RF, Logistic, SMO, Naive Bayes, AdaBoost, AROW, OGD, SCW, PA, Perceptron, IELLIP, ALMA, ROMMA	AROW (test validation)	Error rate 0.190 offline RF ACC 0.981
Sarma [56]	2020	Bangladesh	209	146	63	NR	DT, RF	DT (test validation)	AUC 0.790 ACC 0.790 Sens 0.790 F1-score 0.790
Kapoor [55]	2020	India	101	101	NR	LOOCV for feature	W-ANN, DT, RF, SVM	SVM (internal validation)	ACC 1.000 Error rate 0.000
Huang [54]	2020	Taiwan	798	718	80	Stratified 10-fold	LR, SVM, RF, GBM, ANN	ANN (internal validation)	AUC 0.832 Balanced ACC 0.752
Ho [53]	2020	Taiwan	4894	NR	NR	10-fold CV × 20 repeats	DT, DNN, LR	DNN (internal validation)	AUC 0.859 ACC 0.810 Sens 0.900 Spec 0.674 PPV 0.806
Iqbal [58]	2019	India	75	NR	NR	10-fold	LogitBoost, LR, DT, NB, ANN, SMO/SVM, KNN	LogitBoost (internal validation)	AUC 0.967 ACC 0.920 Sens 0.900 Spec 0.940 Precision 0.950 F1-score 0.920
Davi [57]	2019	Brazil	102	77	25	3-fold for SVM-RFE; stratified 10-fold for ANN	SVM-RFE, ANN	SVM-RFE + ANN (test validation)	AUC 0.928 ACC 0.960 Sens 1.000 Spec 0.857 Precision 0.947 F1-score 0.973
Khan [59]	2016	Pakistan	84	NR	NR	10-fold	SVM-RBF, SVM-polynomial, SVM-linear	SVM-polynomial order 1 (internal validation)	ACC 0.850 Sens 0.730 Spec 0.930 Precision 0.900

ACC, accuracy; AdaBoost, adaptive boosting; ANN, artificial neural network; AROW, adaptive regularization of weights; AUC, area under the receiver operating characteristic curve; Balanced ACC, balanced accuracy; Bi-LSTM, bidirectional long short-term memory; CART, classification and regression tree; CNN, convolutional neural network; CV, cross-validation; DNN, deep neural network; DSS, dengue shock syndrome; DT, decision tree; ETC, Extra Trees classifier; F1-score, harmonic mean of precision and recall; GBC, gradient boosting classifier; GBDT, gradient-boosted decision tree; GBM, gradient boosting machine; GRU, gated recurrent unit; KNN, k-nearest neighbors; Lasso-LR, least absolute shrinkage and selection operator logistic regression; LDA, linear discriminant analysis; LightGBM, Light Gradient Boosting Machine; LOOCV, leave-one-out cross-validation; LR, logistic regression; LSTM, long short-term memory; MARS, multivariate adaptive regression splines; MLP, multilayer perceptron; NB, naive Bayes; NPV, negative predictive value; NR, not reported; PCA-DA, principal component analysis–discriminant analysis; PCA-SVM, principal component analysis–support vector machine; PLS-DA, partial least squares discriminant analysis; PLSR, partial least squares regression; PPV, positive predictive value; RF, Random Forest; RNN, recurrent neural network; ROC, receiver operating characteristic; Sens, sensitivity; SMO, sequential minimal optimization; Spec, specificity; SVC, support vector classifier; SVM, support vector machine; SVM-RBF, support vector machine with a radial basis function kernel; SVM-RFE, support vector machine with recursive feature elimination; TAN, tree-augmented naive Bayes; W-ANN, weight-based artificial neural network; WHO, World Health Organization; XGBoost, Extreme Gradient Boosting.

## Data Availability

No new data were collected or generated for this study. The dataset used in this secondary analysis is publicly available in the Mendeley Data repository, Version 2, at DOI: 10.17632/6fsrsk3mb8.2.

## Supplementary Materials

The following supporting information can be downloaded at: https://www.mdpi.com/article/10.3390/diagnostics16182966/s1, Figure S1: Correlation matrix of the demographic and hematological predictors included in the analysis.
